# Supplementary material for: Phosphatidylethanolamine modulates α-synuclein membrane-binding behavior
Source: Biophys J. 2025 Dec 23;125(3):811–20. doi: 10.1016/j.bpj.2025.12.025 (PMC13351367; doi:10.1016/j.bpj.2025.12.025)
Supplement: Document S1. Figures S1–S6 and Table S1 [file mmc1.pdf]

Biophysical Journal, Volume *125*

Supplemental information

Phosphatidylethanolamine modulates α-synuclein membrane-binding behavior

Norihiro Namba, Shiori Ariyoshi, Honori Shiroshita, Norihisa Yoshimura, Takashi Ohgita, Shinya Oishi, and Hiroyuki Saito

Supporting Information

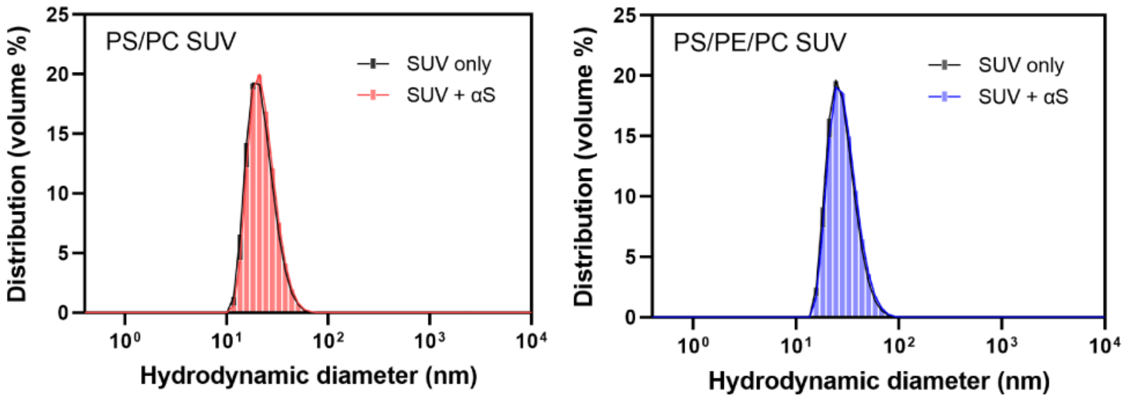

Figure S1. Effect of α-synuclein (αS) addition on particle size distribution of small unilamellar vesicles (SUVs). Dynamic light scattering measurements were performed using a Zetasizer Nano ZS (Malvern) to assess the size distribution of SUVs (phospholipid concentration was 500 μg/mL) in the absence and presence of αS (25 μg/mL). Left panel, PS/PC (3/7) SUVs; Right panel, PS/PE/PC (3/5/2) SUVs. PE, phosphatidylethanolamine; PC, phosphatidylcholine; PS, phosphatidylserine. All data represent the average of three independent measurements.

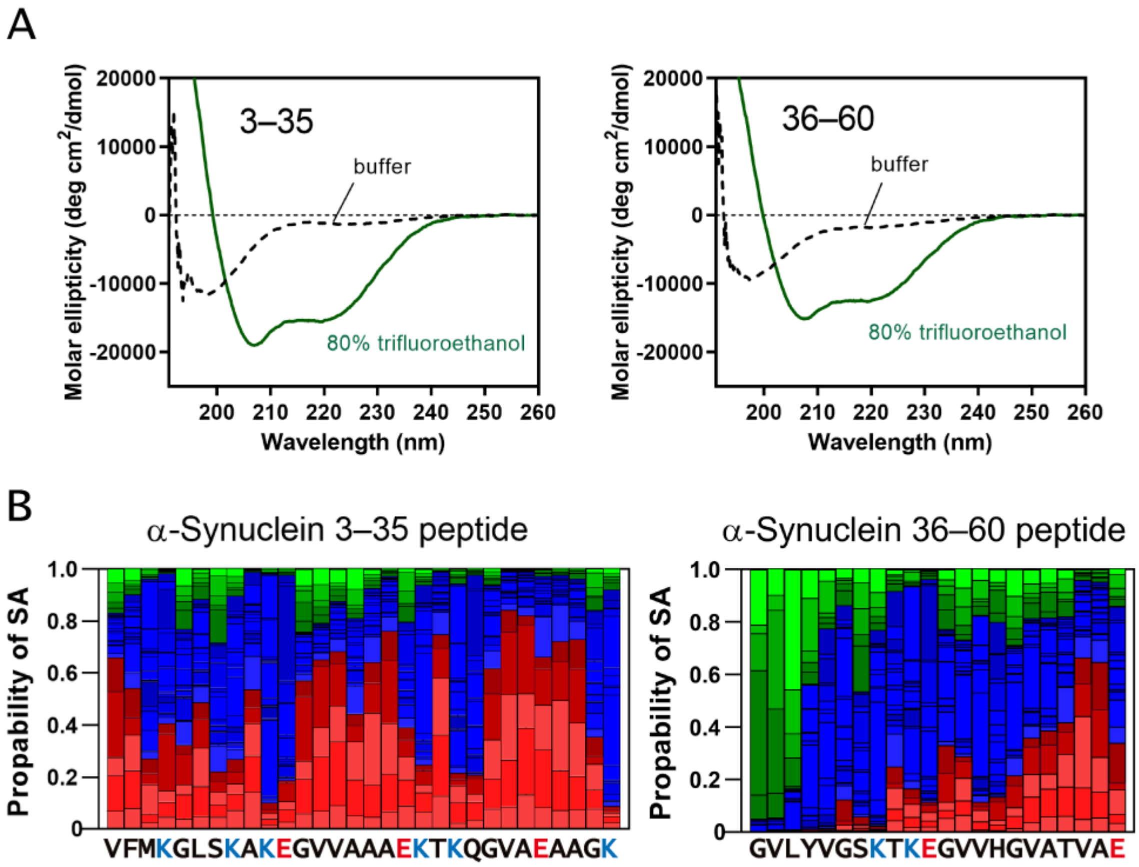

Figure S2. Secondary structure analysis of αS N-terminal peptides. (A) Far-UV circular dichroism (CD) spectra of αS peptides corresponding to residues 3–35 and 36–60 (10 μM) in 80% (v/v) trifluoroethanol/20 mM phosphate buffer (50 mM NaCl, pH 7.4). The spectra indicate their α-helical formation propensity. All data represent the average of at least two independent measurements. (B) Predicted secondary structural propensities of αS 3–35 and 36–60 peptides using the PEP-FOLD4 server (https://bioserv.rpbs.univ-paris-diderot.fr/services/PEP-FOLD4/). SA, structural alphabet. Red, blue, and green bars represent helical, coil, and extended structures, respectively.

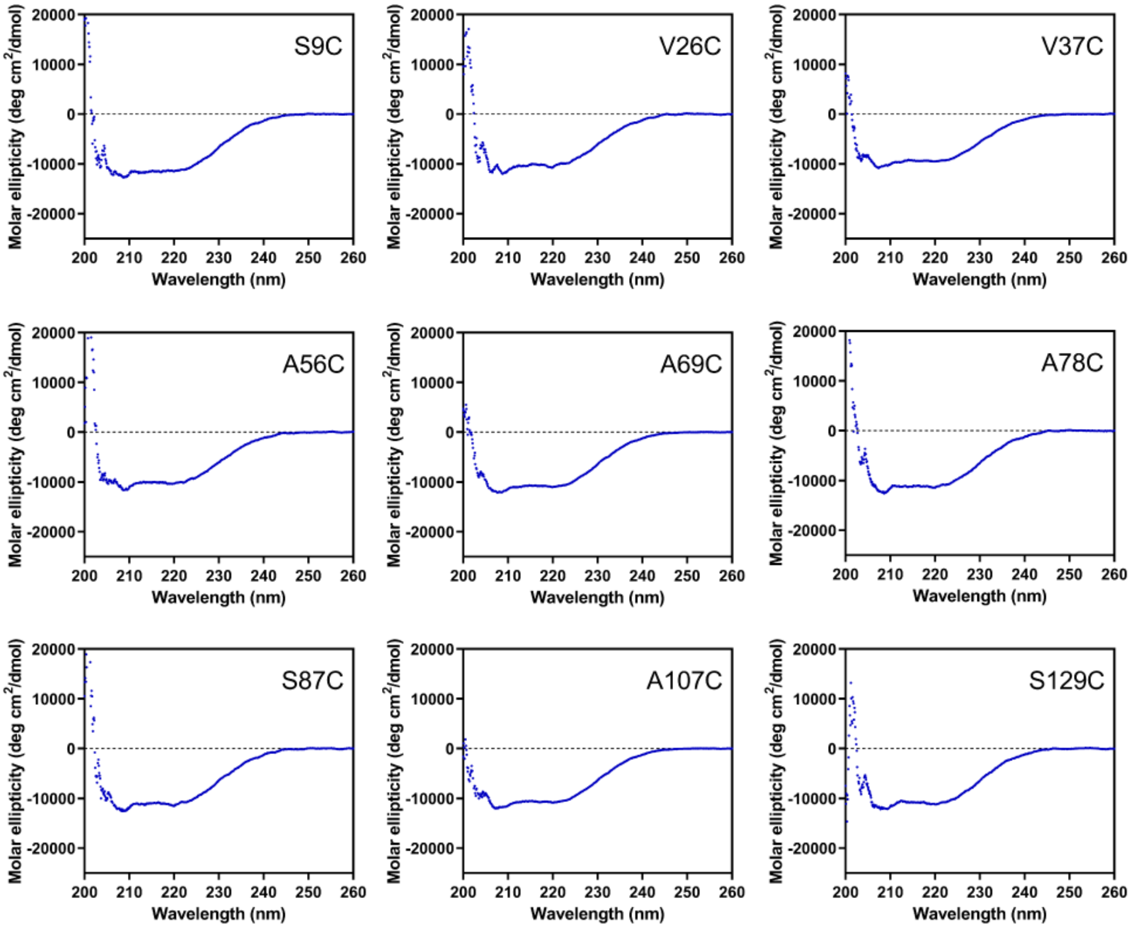

Figure S3. Far-UV CD spectra of Cys-substituted variants of αS. Far-UV CD spectra of each αS variant (50 µg/mL) were recorded in the presence of PS/PC (3/7) SUVs (phospholipid concentration was 1 mg/mL) in 20 mM phosphate buffer (150 mM NaCl, pH 7.4) using a JASCO J-1500 spectropolarimeter.

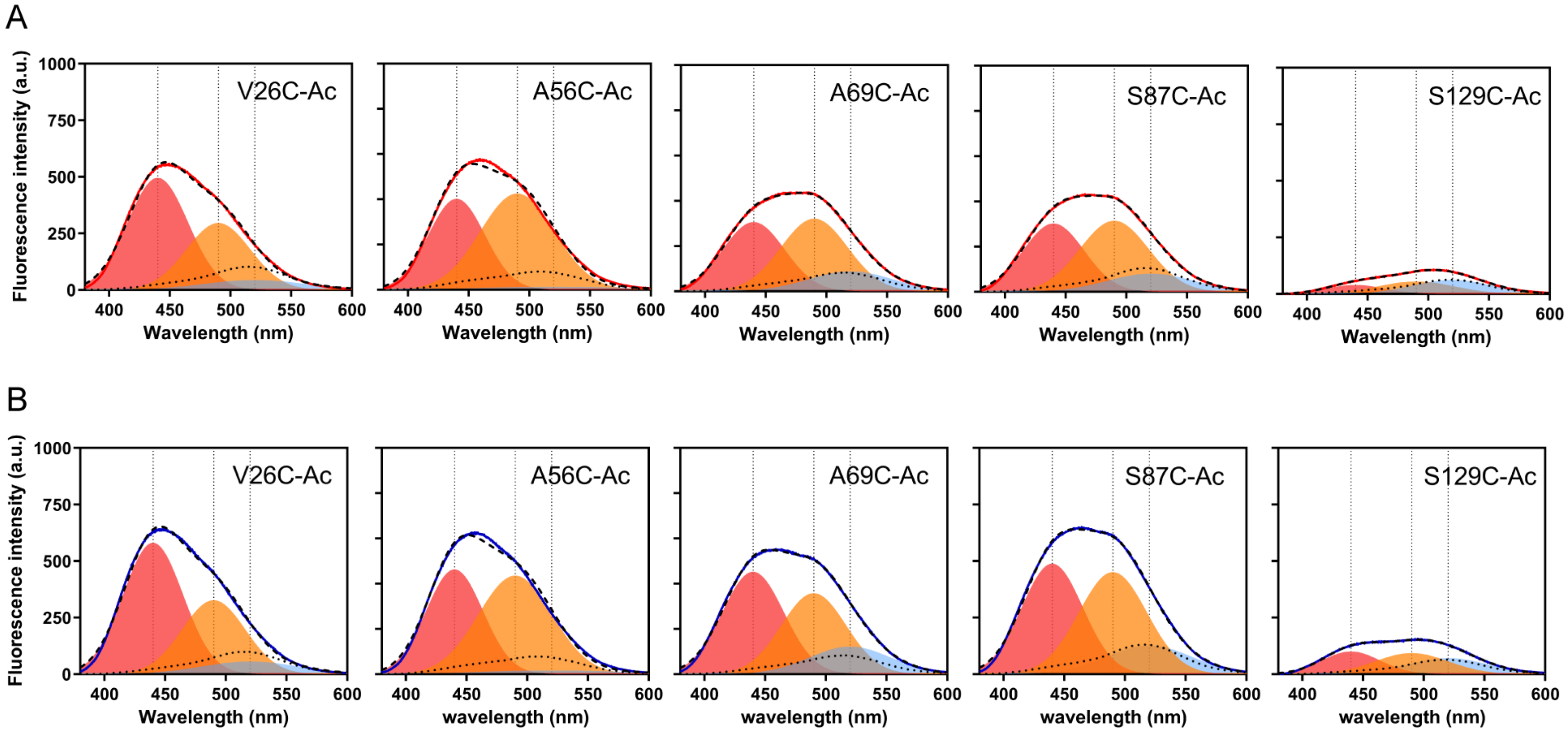

Figure S4. Gaussian deconvolution of acrylodan (Ac) fluorescence spectra of Ac-labelled αS variants. Ac fluorescence spectra of αS variants (25 μg/mL) were recorded in the presence of PS/PC (3/7) SUVs (A) and PS/PE/PC (3/5/2) SUVs (B) in 20 mM phosphate buffer (150 mM NaCl, pH 7.4) using an F-7000 fluorescence spectrophotometer (Hitachi High-Tech). The phospholipid concentration was 500 μg/mL. Spectra were deconvoluted into three Gaussian components centered at 440 nm (red), 490 nm (orange), and 520 nm (blue) using the least square method. All data represent the average of at least three independent measurements.

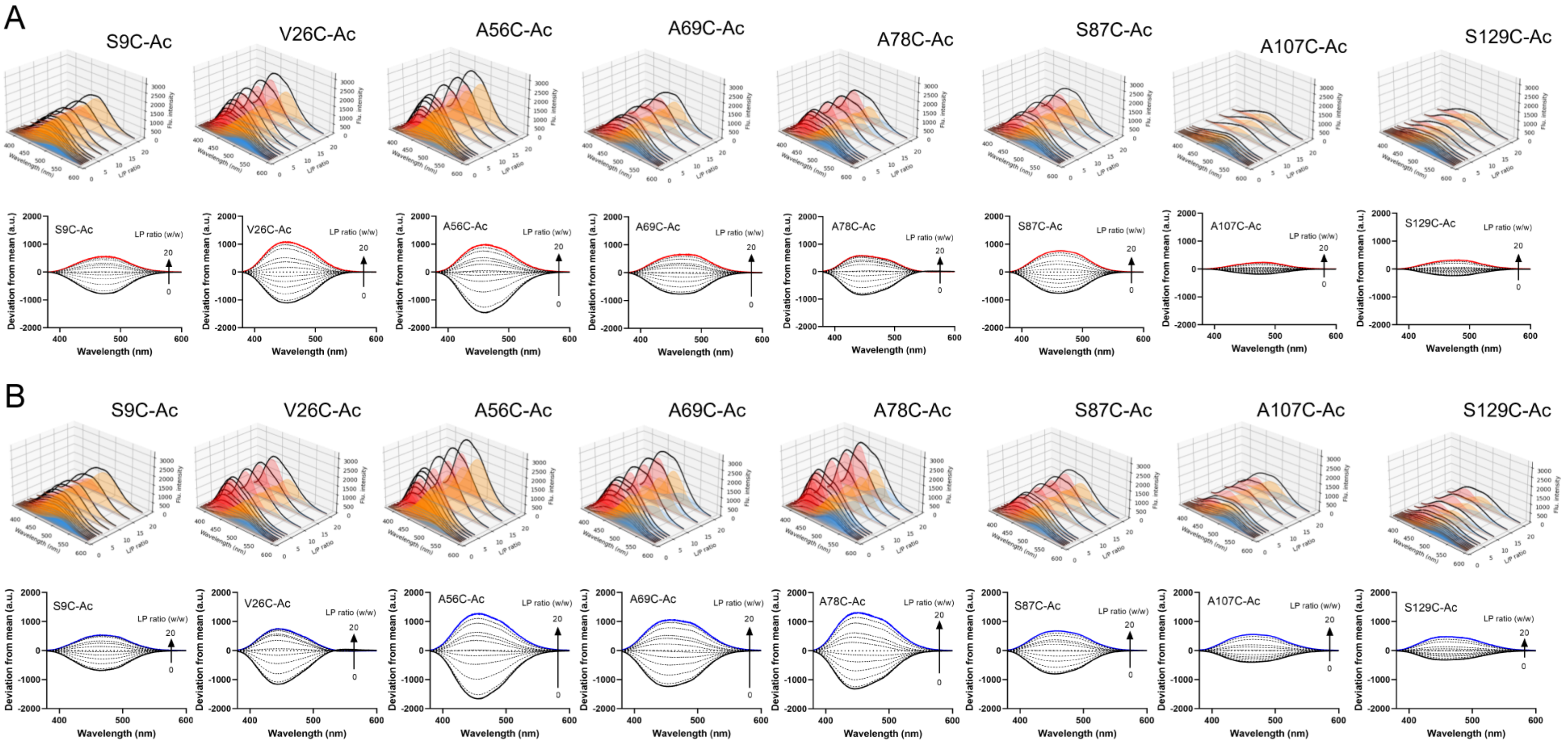

Figure S5. Ac fluorescence spectra of αS variants labeled at individual residues with varying phospholipid-to-protein ratios. Spectra were recorded in the presence of PS/PC (3/7) SUVs (A) and PS/PE/PC (3/5/2) SUVs (B) in 20 mM phosphate buffer (150 mM NaCl, pH 7.4). Upper panels, Ac fluorescence spectra of each Ac-labelled αS variant at different phospholipid-to-protein ratios. Lower panels, Mean-centered spectra used for principal component analysis (PCA). The measurements were performed at least twice for each experimental condition.

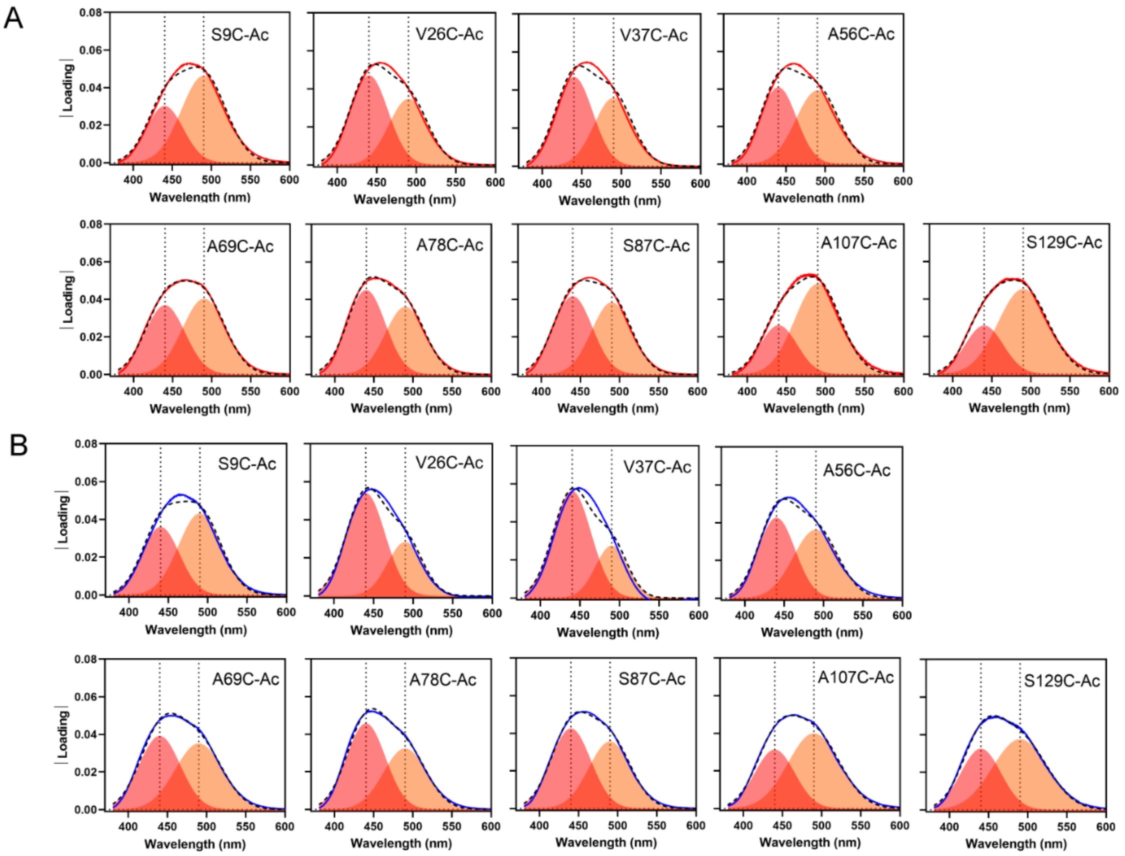

Figure S6. Gaussian deconvolution of first principal component (PC1) loading spectra of Ac-labeled αS variants. PC1 loading spectra were derived using PCA of mean-centered Ac fluorescence spectra (see Figure S5), obtained under conditions using SUVs composed of PS/PC (3/7) (A) and PS/PE/PC (3/5/2) (B). Dotted and solid lines represent PC1 loading spectra and the results of Gaussian deconvolution, respectively. Red- and orange-shaded areas correspond to Gaussian components with maxima at 440 nm and 490 nm, respectively.

Table S1. Contribution ratios of the top five components derived using PCA. The table presents a summary of the proportion of total variance (normalized to 1.0) explained by the first five principal components (PC1–PC5) obtained using PCA of Ac-fluorescence spectra. PC, principal component.

PS/PC (3/7)

	**S9C**	**V26C**	**V37C**	**A56C**	**A69C**	**A78C**	**S87C**	**A107C**	**S129C**
**PC1**	0.9998	0.9994	0.9994	0.9994	0.9988	0.9992	0.9996	0.9993	0.9996
**PC2**	0.0001	0.0004	0.0005	0.0004	0.0011	0.0008	0.0004	0.0003	0.0003
**PC3**	1.9E–5	0.0001	7.1E–5	0.0002	9.5E–6	2.7E–5	4.2E–6	6.9E–5	3.7E–5
**PC4**	1.1E–5	5.5E–6	5.6E–6	5.5E–6	8.8E–6	5.4E–6	2.6E–6	5.9E–5	2.9E–5
**PC5**	9.2E–6	4.7E–6	4.1E–6	4.8E–6	7.5E–6	5.0E–6	1.6E–6	4.6E–5	2.2E–5

PS/PE/PC (3/5/2)

	**S9C**	**V26C**	**V37C**	**A56C**	**A69C**	**A78C**	**S87C**	**A107C**	**S129C**
**PC1**	0.9997	0.9994	0.9993	0.9989	0.9976	0.9974	0.9989	0.9998	0.9998
**PC2**	0.0002	0.0006	0.0006	0.0010	0.0024	0.0026	0.0011	0.0001	0.0002
**PC3**	1.7E–5	1.4E–5	9.8E–6	0.0001	7.6E–6	7.4E–6	3.5E–6	2.6E–5	1.9E–5
**PC4**	1.2E–5	7.0E–6	7.1E–6	4.2E–6	6.3E–6	4.4E–6	2.4E–6	5.9E–6	1.5E–5
**PC5**	1.0E–5	5.9E–6	6.0E–6	3.7E–6	5.7E–6	3.4E–6	1.7E–6	3.3E–6	1.3E–5
