# Supplementary material for: Phosphatidylethanolamine modulates α-synuclein membrane-binding behavior
Source: Biophys J. 2025 Dec 23;125(3):811–20. doi: 10.1016/j.bpj.2025.12.025 (PMC13351367; doi:10.1016/j.bpj.2025.12.025)
Supplement: Document S2. Article plus supporting material [file mmc2.pdf]

Biophysical Journal
Article

Biophysical Society

# Phosphatidylethanolamine modulates α-synuclein membrane-binding behavior

Norihiro Namba,[1] Shiori Ariyoshi,[1] Honori Shiroshita,[1] Norihisa Yoshimura,[1] Takashi Ohgita,[2,*] Shinya Oishi,[3] and Hiroyuki Saito[1]

[1]Laboratory of Biophysical Chemistry, Kyoto Pharmaceutical University, 5 Misasagi-Nakauchi-cho, Yamashina-ku, Kyoto 607-8414, Japan; [2]Center for Instrumental Analysis, Kyoto Pharmaceutical University, 1 Misasagi-Shichono-cho, Yamashina-ku, Kyoto 607-8412, Japan; and [3]Laboratory of Medicinal Chemistry, Kyoto Pharmaceutical University, 1 Misasagi-Shichono-cho, Yamashina-ku, Kyoto 607-8412, Japan

ABSTRACT Interaction with lipid membranes is important in the physiological and pathological functioning of α-synuclein (αS) in brain neuronal cells. In this study, we investigated the effect of lipid composition on the membrane-binding behavior of αS using multiple biophysical techniques. Circular dichroism measurement revealed that, although negatively charged phospholipids are necessary for αS to bind to small unilamellar vesicles, the presence of phosphatidylethanolamine (PE) significantly enhances α-helical structure formation, specifically within the first 35 αS residues. To obtain residue-level structural insights into the lipid-bound αS conformation, site-directed labeling was performed with acrylodan—an environmentally sensitive fluorophore—at the N-terminal, central non-amyloid β component region, and C-terminal regions after cysteine substitution. Acrylodan fluorescence measurements at varying lipid-to-protein ratios revealed that, in addition to the negatively charged C-terminal region, the non-amyloid β component region adopts a more solvent-exposed lipid-bound conformation than the N-terminal region. Notably, PE induced a more hydrophobic, lipid-bound conformation in the N-terminal region of αS than observed with vesicles lacking PE, whereas it promoted association of the C-terminal region with the membrane surface. Collectively, these findings suggest that both the N-terminal and C-terminal regions contribute to αS binding to PE-containing plasma membranes.

SIGNIFICANCE Phosphatidylethanolamine (PE), a key phospholipid in neuronal membranes, modulates the membrane-binding behavior of α-synuclein (αS), a protein implicated in Parkinson disease (PD). Using biophysical approaches, we show that PE enhances α-helical folding of the N-terminal region and promotes membrane association of the C-terminal region of αS. Given reduced PE levels in PD brains, altered lipid composition may disrupt normal function and promote aggregation of αS. These findings provide mechanistic insight into lipid-dependent regulation of αS function and highlight membrane lipids as potential therapeutic targets in PD.

## INTRODUCTION

α-Synuclein (αS) is an intrinsically disordered 140-residue protein localized at the presynaptic terminals of neurons. Although its physiological functions remain incompletely understood, lipid interactions of αS are implicated in neurotransmitter release by regulating synaptic vesicle clustering and trafficking (1,2). Pathologically, abnormal intracellular accumulation of αS as amyloid fibrils is a hallmark of Parkinson disease (PD) (3). Lipid membranes modulate αS aggregation and fibrillization, and membrane lipids co-aggregate with αS fibrils (4–6). Thus, αS-lipid membrane interactions are critical to both its physiological and pathological functions (7).

αS consists of three distinct regions: a positively charged N-terminal region (residues 1–60), a central hydrophobic non-amyloid β component region (NAC) (residues 61–95), and a negatively charged C-terminal region (residues 96–140) (Fig. 1 *A*). Upon binding to negatively charged lipid membranes, αS transitions from a disordered state to an α-helical conformation. The N-terminal region initiates binding to the membrane, followed by the NAC region; both regions form amphipathic α-helices, whereas the C-terminal region remains largely unstructured and weakly associated with the membrane (8,9). The structural plasticity of the N-terminal and NAC regions enables αS to adopt distinct membrane-bound conformations, such as broken

*Submitted October 24, 2025, and accepted for publication December 19, 2025.*

*Correspondence: ohgita@mb.kyoto-phu.ac.jp

Editor: Sudipta Maiti.

https://doi.org/10.1016/j.bpj.2025.12.025

© 2025 The Author(s). Published by Elsevier Inc. on behalf of Biophysical Society.
This is an open access article under the CC BY license (http://creativecommons.org/licenses/by/4.0/).

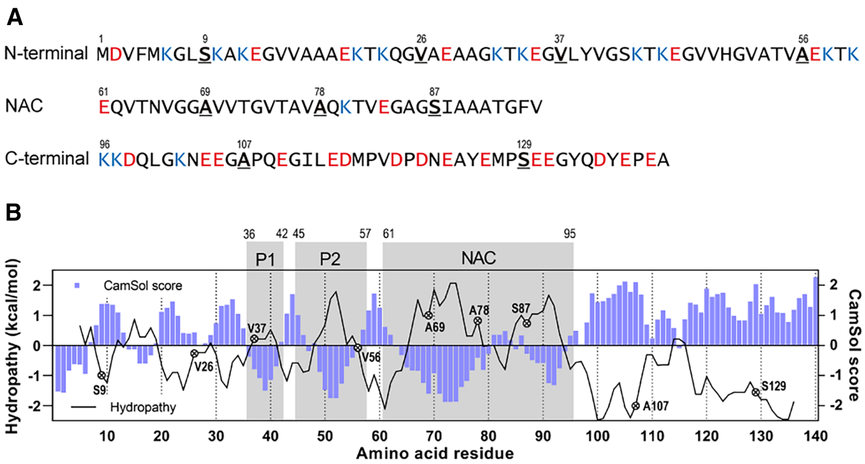


FIGURE 1 Amino acid sequence and hydropathy profile of α-synuclein (αS). (*A*) Amino acid sequence of αS showing its three domains. Positively charged lysine residues are shown in blue, and negatively charged aspartic acid and glutamic acid residues are shown in red. Positions substituted with cysteine for acrylodan labeling are in bold and underlined. NAC, non-amyloid β component region. (*B*) Hydropathy and solubility plots of αS. Hydropathy values were calculated using the ExPASy ProtScale tool (http://web.expasy.org/protscale/) with the Kyte and Doolittle scale and a sliding window of nine residues. Solubility was predicted using the CamSol server (http://www-vendruscolo.ch.cam.ac.uk/camsolmethod.html). Three aggregation-prone regions—P1 (residues 36–42), P2 (residues 45–57), and NAC (residues 61–95) —are highlighted with a gray background.

(10,11) or elongated α-helices (12,13). Furthermore, the surface density of αS, which depends on the lipid-to-protein ratio, has been proposed to regulate its membrane-bound conformation and underlie its aggregation behavior at the membrane surface (14).

Membrane interactions of αS are influenced by several membrane properties, including lipid composition, fluidity, and curvature (15,16). αS preferentially binds to negatively charged lipid membranes through electrostatic interactions involving lysine residues in its N-terminal region (17–19). αS also displays a preference for highly curved membranes, reflecting its affinity for packing defects in lipid bilayers (20–23). Consistently, phospholipids (PLs) with polyunsaturated acyl chains promote αS binding (24), likely because of loose packing resulting from disordered acyl chains. In contrast to interactions with negatively charged PLs such as phosphatidylserine (PS), interactions of αS with zwitterionic PLs are less well characterized. Phosphatidylethanolamine (PE) enhances αS membrane binding (25), whereas phosphatidylcholine (PC) regulates αS-induced membrane tubulation (26,27). In addition, the interactions of amyloidogenic proteins, including αS, with PC vesicles depend on the critical micellar concentration of PC, influencing both amyloid formation and membrane disruption (28). However, how zwitterionic PL composition influences region-specific membrane affinity of αS remains poorly understood.

In this study, we examined the structural transitions of αS and its N-terminal fragment peptides upon binding to small unilamellar vesicles (SUVs) that mimic the composition and curvature of synaptic vesicles. We also analyzed the residue-level membrane-bound conformation of αS on SUVs using site-specific cysteine (Cys) substitution followed by labeling with the environmentally sensitive fluorophore acrylodan (Ac) (29–31) (Fig. 1). Our results indicate that, in addition to the requirement for negatively charged PLs, PE significantly enhances α-helical formation within the first 35 residues. PE also induces a more hydrophobic lipid-bound conformation in the N-terminal region than that observed with vesicles lacking PE and promotes association of the C-terminal region with the membrane surface. These findings suggest that, beyond the N-terminal region, the C-terminal region contributes considerably to αS membrane interactions.

## MATERIALS AND METHODS

### Preparation of recombinant αS proteins and peptides

Recombinant human αS and its Cys-substituted variants were prepared as described previously (32,33). Briefly, αS was expressed in *Escherichia coli* BL21 Star (DE3) as a fusion protein containing an N-terminal thioredoxin and a hexahistidine tag. The fusion protein was purified using Ni-affinity chromatography, followed by proteolytic cleavage of the N-terminal tags using HRV-3C protease. A second round of Ni-affinity chromatography was performed to remove the cleavage tags. The purified αS retained two additional N-terminal residues (Gly-Pro). Protein purity exceeded 95%, as assessed by sodium dodecyl sulfate-polyacrylamide gel electrophoresis and Coomassie Brilliant Blue staining.

Peptides corresponding to αS residues 3–35 and 36–60, and their Trp variants (3–35/F4W and 36–60/V48W), were synthesized using solid-phase peptide synthesis with Fmoc chemistry. The N- and C-termini were capped with acetyl and amide groups, respectively.

### Preparation of SUVs

SUVs were prepared using the lipid film hydration method. PL stock solutions in methanol/chloroform were mixed at the desired molar ratios. The solvent was evaporated using a rotary evaporator to form a thin lipid film, which was then dried overnight in a vacuum desiccator to remove residual solvents. The film was hydrated with 20 mM phosphate buffer (50 or 150 mM NaCl (pH 7.4)) to form a lipid suspension. The suspension was sonicated on ice under nitrogen using a probe-type sonicator until the solution became clear, indicating SUV formation. Insoluble debris and any potential titanium particles released from the ultrasonic probe were removed by ultracentrifugation at 75,000 × $g$ for 1.5 h at 15°C (34,35). The supernatant containing SUVs was collected, and the PC concentration was determined using the Phospholipid C-Test Wako kit (FUJIFILM Wako Chemicals). The averaged hydrodynamic diameters of SUVs were approximately 20 nm, as determined by dynamic light scattering measurements (Fig. S1).

## Circular dichroism measurements

Far-UV circular dichroism (CD) spectra were recorded from 190 to 260 nm at 25°C using a JASCO J-1500 spectropolarimeter (JASCO, Tokyo, Japan) with a 1-mm quartz cuvette. Solutions of αS protein (50 μg/mL) or peptide (10 μM) in 20 mM phosphate buffer (50 or 150 mM NaCl (pH 7.4)) were measured in the absence or presence of SUVs. Spectra were baseline-corrected by subtracting the corresponding blank signal.

The membrane-bound fraction of αS ($f_{\mathrm{bound}}$) was estimated from changes in mean residue ellipticity at 222 nm ($[\theta]_{222}$) as a function of PL/αS ratio using the following equation:

$$f_{\mathrm{bound}} = \left([\theta]_{222} - [\theta]_{222,\mathrm{free}}\right) \Big/ \left([\theta]_{222,\mathrm{plateau}} - [\theta]_{222,\mathrm{free}}\right) \quad \text{(Equation 1)}$$

Here, $[\theta]_{222,\text{ free}}$ and $[\theta]_{222,\text{ plateau}}$ represent mean residue ellipticity values at 222 nm in the absence of SUVs and at the plateau phase of αS membrane binding, respectively. $[\theta]_{222,\text{ plateau}}$ was estimated by fitting the $[\theta]_{222}$ versus PL/αS ratio curve to a one-phase decay model.

The number of lipid molecules involved in αS binding ($1/\sigma$) and the dissociation constant per lipid molecule ($K_d$) were estimated by fitting the $f_{\mathrm{bound}}$ versus lipid concentration curve using the lipid-depletion binding model (36), as given below.

$$f_{\mathrm{bound}} = \left\{(\sigma \cdot K_d + \sigma \cdot L + P) - \sqrt{(\sigma \cdot K_d + \sigma \cdot L + P)^2 - 4K_d \cdot \sigma \cdot P}\right\} \Big/ 2P, \quad \text{(Equation 2)}$$

where, $L$ and $P$ are the total lipid and protein concentrations, respectively.

## Tryptophan fluorescence measurements

Fluorescence spectra of tryptophan (Trp) residues in αS 3–35/F4W and 36–60/V48W peptides were recorded using an F-7000 fluorescence spectrophotometer (Hitachi High-Tech, Tokyo, Japan). Peptide solutions (1 μM) in 20 mM phosphate buffer (50 mM NaCl (pH 7.4)) were measured in the absence or presence of 200 μM SUVs. Emission spectra were collected from 300 to 420 nm with excitation at 280 nm. All spectra were baseline-corrected by subtracting the corresponding blank signal.

## Ac fluorescence measurements

Solutions of αS Cys-substituted variants (10 μM) were incubated with a 10-fold molar excess of tris(2-carboxyethyl)phosphine hydrochloride (TCEP; Pierce, Rockford, IL, USA) and acrylodan (Ac; 6-acryloyl-2-dimethylaminonaphthalene; Molecular Probes, Eugene, OR, USA) in the presence of 2 M urea. The reaction mixture was incubated at 4°C for 48 h in the dark with gentle stirring. Unreacted Ac was removed by repeated dialysis against 20 mM phosphate buffer (150 mM NaCl (pH 7.4)). Labeling efficiency, determined using an extinction coefficient of 19,200 $M^{-1}$ $cm^{-1}$ at 391 nm, ranged from 60% to 100%.

Fluorescence spectra of Ac-labeled αS variants, in the absence or presence of SUVs, were recorded at 25°C using an F-7000 fluorescence spectrophotometer. Emission spectra were collected from 380 to 600 nm with excitation at 360 nm. Each spectrum was deconvoluted into three Gaussian components centered at 440 nm, 490 nm, and 520 nm, corresponding to highly hydrophobic, less hydrophobic, and solvent-exposed environments, respectively. Gaussian fitting was performed via least-squares optimization using the *curve_fit* function from the *scipy.optimize* library in Python. The area under each Gaussian component ($S_{440}$, $S_{490}$, and $S_{520}$) was calculated by numerical integration.

To evaluate the local membrane environment of each Ac-labeled residue, fluorescence spectra obtained at varying PL/protein ratios were mean-centered and subjected to principal component analysis (PCA) using the *scikit-learn* library in Python. The first principal component (PC1), which accounted for more than 99% of the total spectral variance, was extracted for further analysis. PC1 spectra were deconvoluted into two Gaussian components centered at 440 nm and 490 nm. Generalized polarization (GP) values were then calculated using the following equation:

$$\mathrm{GP} = \frac{S_{440} - S_{490}}{S_{440} + S_{490}} \quad \text{(Equation 3)}$$

# RESULTS

## Effect of PE on αS binding to SUVs

To mimic the lipid composition of synaptic vesicles, SUVs composed of 1-palmitoyl-2-oleoyl PS, 1-palmitoyl-2-oleoyl PE, and 1-palmitoyl-2-oleoyl PC (NOF, Tokyo, Japan) were prepared at a 3:5:2 molar ratio (8,37). For comparison, control SUVs containing PS and PC at a 3:7 molar ratio were also used. Dynamic light scattering confirmed that both SUVs exhibited unimodal size distributions with hydrodynamic diameters of approximately 20 nm, which remained unchanged upon αS addition (Fig. S1). These results indicate that αS does not induce significant vesicle deformation under the experimental conditions.

To assess the membrane-induced conformational transition of αS, far-UV CD spectroscopy was performed (Fig. 2 *A*). In the presence of PS-containing SUVs, αS adopted an α-helical conformation, as indicated by characteristic negative ellipticity peaks at 208 and 222 nm. In contrast, PE/PC SUVs produced negligible spectral changes, indicating that negatively charged PS is essential for αS membrane binding and α-helical formation (17–19). Fig. 2 *B* shows changes in mean residue ellipticity at 222 nm with varying PL/αS ratios. Ellipticity decreased more steeply for PS/PE/PC SUVs than for PS/PC SUVs, suggesting that PE enhances α-helical formation upon αS binding to PS-containing SUVs. We note that at the lowest PL concentration (12.5 μg/mL), which is far higher than critical micellar concentration, no significant changes in CD spectra were observed, indicating that the observed interaction of αS occurs with self-assembled vesicles rather than free lipids (28).

To determine thermodynamic parameters of αS-lipid interactions, isothermal adsorption curves derived from ellipticity changes at 222 nm were fitted to a lipid-depletion binding model (36). This model accounts for both direct lipid depletion at protein binding sites and indirect effects such as local lipid rearrangements upon protein binding. The model fit the data well (Fig. 2 *C*), supporting its applicability. The 1/σ values, representing the number of lipid molecules bound per αS molecule, were lower for PS/PE/PC SUVs than for PS/PC SUVs (Fig. 2 *D*). This suggests that replacing PC with PE reduces the lipid contact area per αS molecule, possibly reflecting a more compact membrane-bound conformation that facilitates denser surface accumulation of αS. Furthermore, the dissociation constant

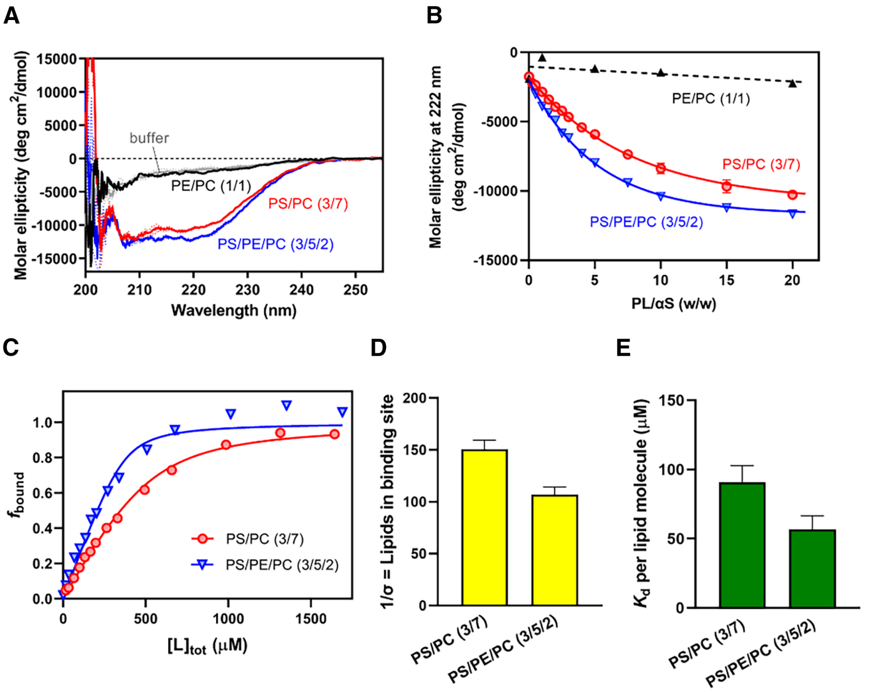


FIGURE 2 Circular dichroism (CD) analysis to evaluate αS membrane binding. (*A*) Far-UV CD spectra of αS (50 μg/mL) in the absence and presence of small unilamellar vesicles (SUVs; PL concentration was 1 mg/mL) in 20 mM phosphate buffer (150 mM NaCl (pH 7.4)). *Deg*, degree of ellipticity. Dotted lines represent standard error (S.E.). (*B*) Changes in molar ellipticity at 222 nm as a function of increasing SUV concentration. Error bars represent S.E. Solid and dotted line graphs indicate nonlinear regression fits to a one-phase decay model. (*C*) Fraction of membrane-bound αS ($f_{bound}$) plotted against total lipid concentration ($[L]_{tot}$). $f_{bound}$ values were calculated from the molar ellipticity at 222 nm. Solid line graphs indicate nonlinear regression fits to the lipid-depletion binding model. Error bars represent S.E. (*D* and *E*) Comparison of $1/\sigma$ values that represent the number of lipid molecules per binding site (*D*) and dissociation constant ($K_d$) per lipid molecule (*E*) between PS/PC (3/7) and PS/PE/PC (3/5/2) SUVs. Values were obtained from the fitting curves in (*C*) using the lipid-depletion binding model. Error bars represent S.E. from curve fitting. PE, phosphatidylethanolamine; PC, phosphatidylcholine; PS, phosphatidylserine.

($K_d$) per lipid molecule was lower for PE-containing SUVs (Fig. 2 *E*), indicating that PE enhances αS binding affinity.

Overall, these findings demonstrate that αS preferentially binds to PE-containing membranes, undergoing a more pronounced α-helical transition and forming a denser accumulation of αS on the membrane surface.

## Effect of PE on the α-helical transition of N-terminal fragment peptides of αS

The N-terminal domain of αS is critical for lipid binding (38,39), and N-terminal acetylation modulates this interaction (40,41). The domain also includes two aggregation-prone regions, designated P1 (residues 36–42) and P2 (residues 45–57) (Fig. 1 *B*) (42). To assess the effect of PE on membrane binding of the N-terminal domain, two peptides corresponding to residues 3–35 and 36–60 of αS were synthesized, and their conformational changes upon SUV binding were analyzed using far-UV CD spectroscopy (Fig. 3 *A* and *B*). Both peptides adopted α-helical conformations in 80% trifluoroethanol (Fig. S2 *A*). Structural propensity predictions using PEP-FOLD4, a fragment-based peptide structure prediction tool (43,44), indicated that the 3–35 peptide has high α-helical propensity, whereas the 36–60 peptide exhibits much less α-helical propensity and favors β-structure formation (Fig. S2 *B*).

Far-UV CD spectra showed that the 3–35 peptide underwent a distinct α-helical transition upon binding to both PS/PE/PC and PS/PC SUVs, as evidenced by characteristic negative peaks at 208 and 222 nm (Fig. 3 *A*). The greater change in molar ellipticity at 222 nm in the presence of PS/PE/PC SUVs than in the presence of PS/PC SUVs suggests that the 3–35 segment preferentially binds to PE-containing vesicles with adopting an α-helical conformation. Consistently, a considerable blue shift in the fluorescence peak of the Trp residue substituted at position F4 in the 3–35 peptide was observed for both SUV types (Fig. 3 *C*). In contrast, the 36–60 peptide did not exhibit significant spectral changes in either far-UV CD or Trp fluorescence spectra upon SUV addition (Fig. 3 *B* and *D*), indicating that this fragment exhibits weak lipid-binding affinity under the experimental conditions.

## Evaluation of site-specific membrane interaction of αS using Ac fluorescence

To investigate how PE affects the local structural environment of lipid-bound αS, we introduced site-specific Cys substitutions into αS and labeled the substituted residues with the environment-sensitive fluorophore Ac (Fig. 1). Far-UV CD spectroscopy confirmed that Cys substitution minimally affected the α-helical transition of αS upon SUV binding (Fig. S3).

In the absence of SUVs, all Ac-labeled αS variants exhibited weak, unimodal fluorescence emission peaks centered at 520 nm, indicating that Ac resides in a high-dielectric aqueous environment (Figs. 4 *A*, *B*, and S4, represented as dotted lines). Upon addition of SUVs at a PL/αS ratio of 20:1 (w/w), each variant displayed distinct spectral shifts. The spectra were consistently deconvoluted into two unimodal components centered at 440 nm and 490 nm (Figs. 4 *A*, *B*, and S4), consistent with previous reports on DAN

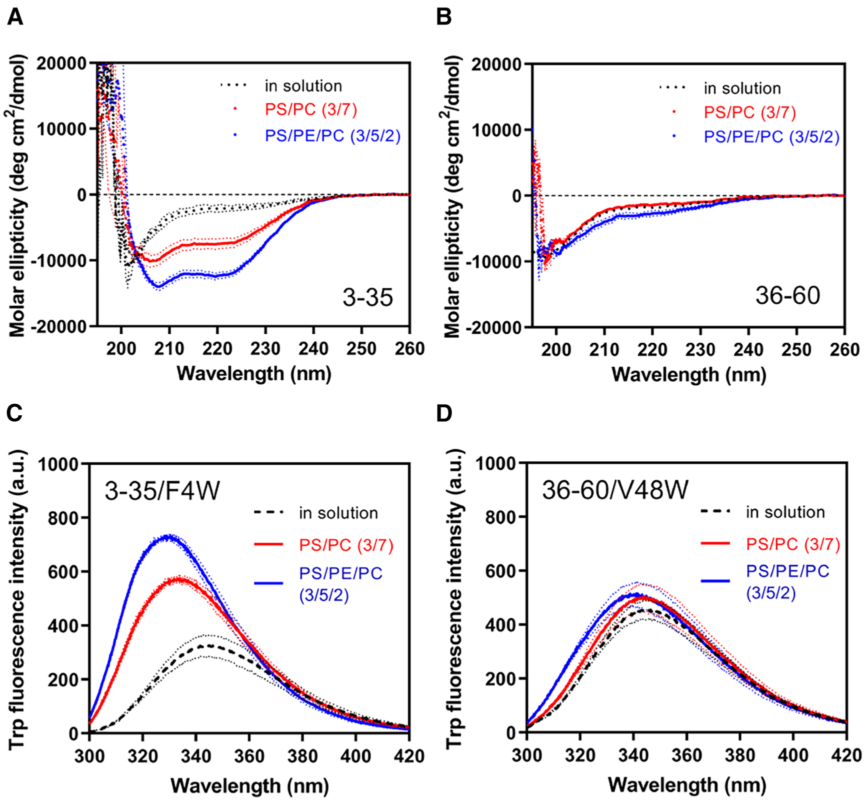


FIGURE 3 Interaction of N-terminal αS fragment peptides with SUVs. (*A* and *B*) Far-UV CD spectra of αS 3–35 (*A*) and 36–60 (*B*) peptides (10 μM) in the absence and presence of SUVs (PL concentration was 2 mM) in 20 mM phosphate buffer (50 mM NaCl (pH 7.4)). *Deg*, degree of ellipticity. (*C* and *D*) Trp fluorescence spectra of αS 3–35/F4W (*C*) and 36–60/V48W (*D*) peptides (1 μM) in the absence and presence of SUVs (200 μM). a.u., arbitrary unit. All data represent the average of at least two independent measurements. Dotted lines indicate standard error from replicate measurements.

fluorophores (45,46). Given that Ac emission shifts to shorter wavelengths in lower dielectric environments (47), the 440-nm component likely corresponds to Ac buried within the hydrophobic environment of the lipid bilayer, whereas the 490-nm component corresponds to Ac positioned in the less hydrophobic membrane surface.

In the presence of PS/PC SUVs, the 520-nm component accounted for a large peak area at residues 107 and 129 (Fig. 4 *C* and *E*), suggesting that the negatively charged C-terminal region remains largely solvent exposed. In contrast, the peak area of the solvent-exposed component decreased in the presence of PS/PE/PC SUVs (Fig. 4 *D* and *E*), indicating that PE promotes membrane association of the C-terminal region.

## Mapping residue-specific membrane interaction of αS via PCA of Ac fluorescence spectra

We next analyzed Ac fluorescence spectra of αS variants across varying PL/αS ratios by applying PCA (Figs. 5 *A* and S5) to determine whether the 440-nm and 490-nm fluorescence components in Ac-labeled αS represent distinct binding states or a single averaged membrane environment (48,49). In all αS variants, spectral changes were explained by a single principal component accounting for >99.7% of total variance (Table S1), indicating that the two components vary cooperatively and reflect a single averaged membrane environment.

To characterize the local membrane environment of each residue in αS, the major principal component spectra were deconvoluted into two Gaussian components centered at 440 nm and 490 nm (Figs. 5 *B* and S6). Relative contributions of more hydrophobic (440 nm) and less hydrophobic (490 nm) environments were quantified as GP values, generating a residue-specific profile of membrane interaction of αS (Fig. 5 *C*). A low GP value at residue 9 indicated less hydrophobic membrane interaction of the N-terminus, consistent with previous findings that non-acetylated N-terminus has a weak membrane interaction and the N-terminal acetylation enhances lipid binding of residues 1–15 (50). This suggests a weak interaction of the non-acetylated N-terminus. Similarly, residues 107 and 129 exhibited low GP values, consistent with their minimal spectral shifts. In contrast, residues 26, 37, 56, 69, 78, and 87 showed relatively high GP values, indicating more hydrophobic membrane interaction from the N-terminal to the NAC regions.

Notably, the presence of PE increased GP values for residues 9–37 in the N-terminal region, suggesting that PE promotes a more ordered and/or more hydrophobic environment around these residues (51). In contrast, GP values for residues 56, 69, 78, and 87 were largely unaffected, indicating that PE does not significantly alter the local environment of the NAC region. In addition, GP values at residues 107 and 129 increased in the presence of PE, suggesting that PE enhances C-terminal interaction with the membrane.

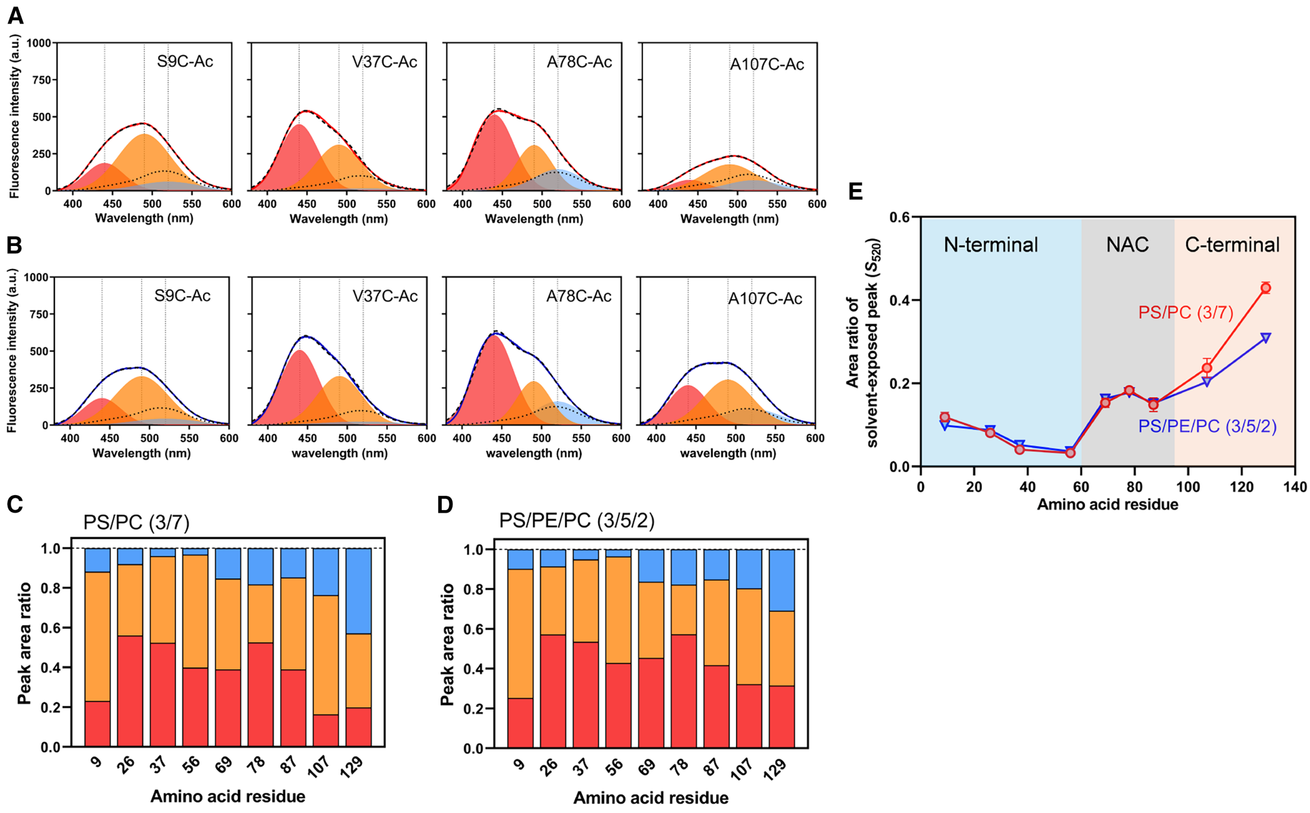


FIGURE 4 Acrylodan (Ac) fluorescence assay to evaluate the residue-specific environment of αS in the presence of SUVs. (*A* and *B*) Ac fluorescence spectra of Cys-substituted αS variants (25 μg/mL) in the presence of PS/PC (3/7) (*A*) or PS/PE/PC (3/5/2) (*B*) SUVs (PL concentration was 500 μg/mL) in 20 mM phosphate buffer (150 mM NaCl (pH 7.4)). The Ac spectra in the absence of SUVs are shown in black dotted lines. Dashed and solid lines indicate measured and Gaussian-fitted spectra, respectively. Red, orange, and blue shaded areas correspond to Gaussian components with maxima at 440 nm (highly hydrophobic), 490 nm (less hydrophobic), and 520 nm (solvent exposed), respectively. Spectra for additional Cys-substituted variants are shown in Fig. S4. (*C* and *D*) Peak area ratios of each Gaussian component in the presence of PS/PC (3/7) (*C*) and PS/PE/PC (3/5/2) (*D*) SUVs. Red, orange, and blue bars correspond to Gaussian components with maxima at 440 nm, 490 nm, and 520 nm, respectively. (*E*) Mapping of the area ratio of solvent-exposed peak ($S_{520}$) as a function of αS residue number. All data represent the average of at least three independent measurements.

Collectively, these results demonstrate that PE modulates the membrane interaction of both the N- and C-terminal regions of αS, whereas the environment of the NAC region remains largely unchanged.

## DISCUSSION

Lipidomic analyses of PD brains have revealed a marked decrease in PE and a concomitant increase in PS levels (52,53). In *Saccharomyces cerevisiae* and *Caenorhabditis elegans*, reduced PE levels lead to αS accumulation and foci formation, mediated by endoplasmic reticulum stress and impaired vesicle trafficking (54). These findings suggest a mechanistic link between PE deficiency and αS dysfunction, potentially contributing to PD pathogenesis.

### Membrane-bound conformation of αS

In this study, we investigated the membrane-bound conformation of αS and its modulation by PE using a suite of physicochemical approaches. CD spectroscopy confirmed that αS binds to PS-containing lipid vesicles and adopts an α-helical structure (Fig. 2), consistent with previous reports (17–19). Using fragment peptides corresponding to the N-terminal region of αS, we found that residues 3–35 form an α-helix upon SUV binding, whereas residues 36–60 largely remain unstructured (Fig. 3), supporting the role of the N-terminal 3–35 segment as a membrane anchor (8,39). Ac fluorescence assays combined with PCA revealed that in PS/PC SUVs, approximately the first 10 residues reside in a less hydrophobic environment, whereas residues 26–87 occupy a more hydrophobic environment (Fig. 5 *C*). This suggests that the aggregation-prone P1 (residues 36–42), P2 (residues 45–57), and NAC regions (Fig. 1 *B*) are less solvent exposed, which may reduce their aggregation propensity. In contrast, the C-terminal region remained largely solvent exposed, consistent with its limited membrane interaction (Fig. 4 *E*).

The discrepancy between the lack of structural transition and membrane binding in the 36–60 peptide (Fig. 3 *B* and *D*) and the spectral changes in Ac spectra observed at residues 37 and 56 in full-length αS in the presence of SUVs (Figs. 4

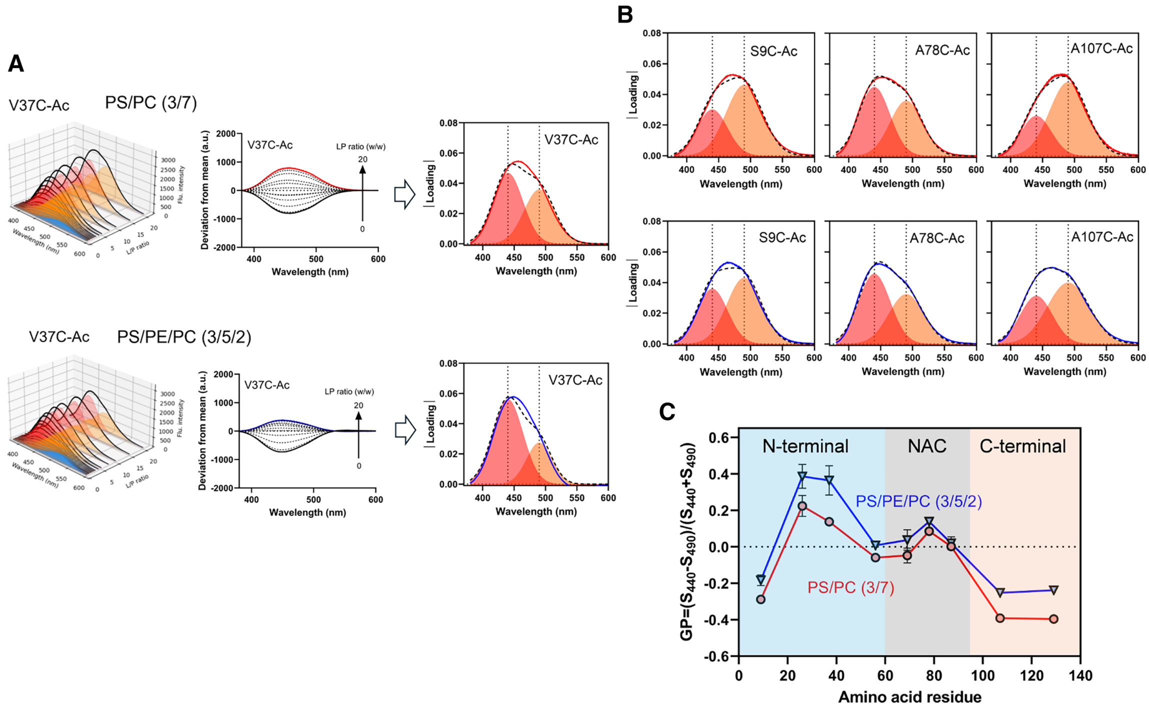


FIGURE 5 Principal component analysis (PCA) of Ac fluorescence spectra at varying SUV concentrations. (*A*) Schematic of PCA applied to Ac fluorescence spectra of the αS V37C-Ac variant obtained at varying phospholipid/protein ratios (*left*). Mean-centered spectra (*middle*) were subjected to PCA. Data for other variants are shown in Fig. S5. The first principal component (PC1) accounted for more than 99% of the total spectral variance for all Cys-substituted αS variants (Table S1). PC1 spectra were deconvoluted into two Gaussian components centered at 440 nm and 490 nm (*right*). Dotted and solid lines represent PC1 loading spectra and Gaussian fits, respectively. Red- and orange-shaded areas correspond to Gaussian components with maxima at 440 nm and 490 nm, respectively. (*B*) Gaussian-deconvoluted PC1 loading spectra of the S9C-Ac, A78C-Ac, and A107C-Ac variants. Data for all Cys-substituted αS variants are shown in Fig. S6. (*C*) Mapping of generalized polarization (GP) values as a function of αS residue number. GP values were calculated as $GP = (S_{440} - S_{490})/(S_{440} + S_{490})$, where $S_{440}$ and $S_{490}$ are the peak areas of the Gaussian components of PC1 loading centered at 440 nm and 490 nm, respectively. The N-terminal, NAC, and C-terminal regions are highlighted in blue, gray and orange, respectively. The measurements were performed at least twice for each experimental condition.

and 5) can be explained by the "initiation–elongation" model (50,55). According to this model, residues 6–25 serve as a membrane anchor (8,39), and α-helical folding subsequently propagates toward the NAC region (50,55). Thus, membrane binding initiated by the N-terminal 3–35 segment enables association of residues 36–60 within the full-length protein, despite their intrinsically low lipid affinity and β-sheet propensity (Fig. S2 *B*).

## Effect of PE on the membrane interaction of αS

Incorporating PE into PS/PC membranes enhanced α-helical folding of full-length αS and its 3–35 residue peptide (Figs. 2 and 3). Consistently, PE increased GP values at residues 9, 26, and 37, particularly at 26 and 37, suggesting a more hydrophobic environment surrounding these residues in PE-containing vesicles. In contrast, PE had little effect on GP values at residues 56, 69, 78, and 87, indicating minimal influence on the NAC region (Fig. 5 *C*). The cone-shaped geometry of PE introduces packing defects in highly curved membranes such as SUVs, transiently exposing hydrophobic regions of the bilayer. αS preferentially binds to such defect-rich vesicles, inserting its hydrophobic side chains into the exposed acyl core (20–22,56). Therefore, the enhanced hydrophobic interactions of the N-terminal region observed by Ac fluorescence measurements likely reflect increased contacts between the amphipathic N-terminal α-helix and the solvent-exposed hydrophobic core of the membrane created by PE.

These PE-dependent hydrophobic interactions partially align with a previous NMR study proposing residues 26–98 as a "membrane sensor" (8). Although direct conformational changes were not observed using NMR, a functional involvement was inferred for this segment based on signal perturbations, detected using a combination of solution and solid-state NMR techniques, in flanking regions (residues 1–25 and 99–140) (8). In contrast, the Ac fluorescence assay in our study directly captured local environmental changes, pinpointing residues around 26–37 as key PE sensors. Differential PE sensitivity between the N-terminal and NAC regions may allow these segments to bind with either the same or different vesicles—a "double anchoring" mechanism proposed to regulate neurotransmitter release (8,57,58). Additionally, the relatively weak hydrophobic interactions of the NAC region compared with those of the N-terminal region on PS/PE/PC SUVs supports a model in

which high αS concentrations promote an upright NAC orientation, facilitating nucleation (14). At elevated αS concentrations, preferential N-terminal interactions with membranes may displace the NAC region from the membrane surface, resulting in an upright, solvent-exposed conformation. These findings highlight how lipid composition and αS surface density cooperate to regulate the protein's membrane-bound conformation.

The Ac fluorescence data obtained in our study also revealed that PE promotes membrane interactions of C-terminal residues 107 and 129 (Fig. 4). This agrees with recent findings that hydrophobic residues I112, L113, and P117 within the C-terminal region can embed into membranes without forming α-helices. Similarly, Y125 and M127 insert deeply into membranes under low-salt conditions, shielding aggregation-prone Tyr rings, thereby suppressing αS fibrillation. However, these interactions weaken at physiological NaCl concentrations (59). Moreover, calcium ions enhance C-terminal binding to synaptic vesicles, modulating vesicle clustering (60). Thus, reduced PE levels in PD could weaken these interactions, perturbing vesicle homeostasis, impairing neurotransmitter release, and promoting αS aggregation.

## CONCLUSION

In summary, the study demonstrated that PE enhances the membrane interactions of αS through its effects on both the N- and C-terminal regions, thereby modulating the structural dynamics and functional states of membrane-bound αS. Given the reduction of PE levels in PD brains, such alterations may promote abnormal synaptic vesicle clustering, impaired neurotransmitter release, and pathological aggregation. These findings underscore the crucial role of membrane lipid composition in regulating αS behavior and suggest that modulating lipid environments could represent a novel and promising therapeutic avenue for PD.

## DATA AND CODE AVAILABILITY

All data generated or analyzed in this study are included in this article and its Supporting Material files.

## ACKNOWLEDGMENTS

This work was partially supported by JSPS KAKENHI (grant numbers JP25K09932 (H.S.) and JP24K09756 (T.O.)) and a Nagai Memorial Research Scholarship from the Pharmaceutical Society of Japan (N.N.). We thank Dr. Keisuke Aoki (Kyoto University) and Mr. Shuma Mitasaki (Kyoto Pharmaceutical University) for technical assistance with peptide preparation.

## AUTHOR CONTRIBUTIONS

N.N., T.O., and H.S. designed the study. N.Y. and S.O. synthesized the peptides. N.N., S.A., and H.S. performed all of the other experiments. N.N., T.O., and H.S. analyzed data and prepared the manuscript. All of the authors reviewed the results and approved the final version of the manuscript.

## DECLARATION OF INTERESTS

The authors declare no competing interests.

## SUPPORTING MATERIAL

Supporting material can be found online at https://doi.org/10.1016/j.bpj.2025.12.025.

## REFERENCES

1. Sharma, M., and J. Burre. 2023. alpha-Synuclein in synaptic function and dysfunction. *Trends Neurosci.* 46:153–166. https://doi.org/10.1016/j.tins.2022.11.007.
2. Diao, J., J. Burré, …, A. T. Brunger. 2013. Native alpha-synuclein induces clustering of synaptic-vesicle mimics via binding to phospholipids and synaptobrevin-2/VAMP2. *eLife.* 2:e00592. https://doi.org/10.7554/eLife.00592.
3. Calabresi, P., A. Mechelli, …, V. Ghiglieri. 2023. Alpha-synuclein in Parkinson's disease and other synucleinopathies: from overt neurodegeneration back to early synaptic dysfunction. *Cell Death Dis.* 14:176. https://doi.org/10.1038/s41419-023-05672-9.
4. Fanning, S., D. Selkoe, and U. Dettmer. 2020. Parkinson's disease: proteinopathy or lipidopathy? *npj Parkinson's Dis.* 6:3. https://doi.org/10.1038/s41531-019-0103-7.
5. Sant, V., D. Matthes, …, L. B. Andreas. 2025. Lipidic folding pathway of alpha-Synuclein via a toxic oligomer. *Nat. Commun.* 16:760. https://doi.org/10.1038/s41467-025-55849-3.
6. Galvagnion, C., A. K. Buell, …, C. M. Dobson. 2015. Lipid vesicles trigger alpha-synuclein aggregation by stimulating primary nucleation. *Nat. Chem. Biol.* 11:229–234. https://doi.org/10.1038/nchembio.1750.
7. Alza, N. P., P. A. Iglesias González, …, G. A. Salvador. 2019. Lipids at the Crossroad of alpha-Synuclein Function and Dysfunction: Biological and Pathological Implications. *Front. Cell. Neurosci.* 13:175. https://doi.org/10.3389/fncel.2019.00175.
8. Fusco, G., A. De Simone, …, G. Veglia. 2014. Direct observation of the three regions in alpha-synuclein that determine its membrane-bound behaviour. *Nat. Commun.* 5:3827. https://doi.org/10.1038/ncomms4827.
9. Wang, C., C. Zhao, …, C. Liu. 2016. Versatile Structures of alpha-Synuclein. *Front. Mol. Neurosci.* 9:48. https://doi.org/10.3389/fnmol.2016.00048.
10. Chandra, S., X. Chen, …, T. C. Südhof. 2003. A broken alpha -helix in folded alpha -Synuclein. *J. Biol. Chem.* 278:15313–15318. https://doi.org/10.1074/jbc.M213128200.
11. Lokappa, S. B., and T. S. Ulmer. 2011. Alpha-synuclein populates both elongated and broken helix states on small unilamellar vesicles. *J. Biol. Chem.* 286:21450–21457. https://doi.org/10.1074/jbc.M111.224055.
12. Jao, C. C., B. G. Hegde, …, R. Langen. 2008. Structure of membrane-bound alpha-synuclein from site-directed spin labeling and computational refinement. *Proc. Natl. Acad. Sci. USA.* 105:19666–19671. https://doi.org/10.1073/pnas.0807826105.
13. Georgieva, E. R., T. F. Ramlall, …, D. Eliezer. 2008. Membrane-bound alpha-synuclein forms an extended helix: long-distance pulsed ESR measurements using vesicles, bicelles, and rodlike micelles. *J. Am. Chem. Soc.* 130:12856–12857. https://doi.org/10.1021/ja804517m.
14. Roeters, S. J., K. Strunge, …, T. Weidner. 2023. Elevated concentrations cause upright alpha-synuclein conformation at lipid interfaces. *Nat. Commun.* 14:5731. https://doi.org/10.1038/s41467-023-39843-1.

15. Pfefferkorn, C. M., Z. Jiang, and J. C. Lee. 2012. Biophysics of alpha-synuclein membrane interactions. *Biochim. Biophys. Acta.* 1818:162–171. https://doi.org/10.1016/j.bbamem.2011.07.032.

16. Makasewicz, K., S. Linse, and E. Sparr. 2024. Interplay of alpha-synuclein with Lipid Membranes: Cooperative Adsorption, Membrane Remodeling and Coaggregation. *JACS Au.* 4:1250–1262. https://doi.org/10.1021/jacsau.3c00579.

17. Viennet, T., M. M. Wördehoff, …, M. Etzkorn. 2018. Structural insights from lipid-bilayer nanodiscs link alpha-Synuclein membrane-binding modes to amyloid fibril formation. *Commun. Biol.* 1:44. https://doi.org/10.1038/s42003-018-0049-z.

18. Pirc, K., and N. P. Ulrih. 2015. alpha-Synuclein interactions with phospholipid model membranes: Key roles for electrostatic interactions and lipid-bilayer structure. *Biochim. Biophys. Acta.* 1848:2002–2012. https://doi.org/10.1016/j.bbamem.2015.06.021.

19. Andersson, A., S. Linse, …, P. Jönsson. 2024. The density of anionic lipids modulates the adsorption of alpha-Synuclein onto lipid membranes. *Biophys. Chem.* 305:107143. https://doi.org/10.1016/j.bpc.2023.107143.

20. Varkey, J., J. M. Isas, …, R. Langen. 2010. Membrane curvature induction and tubulation are common features of synucleins and apolipoproteins. *J. Biol. Chem.* 285:32486–32493. https://doi.org/10.1074/jbc.M110.139576.

21. Ouberai, M. M., J. Wang, …, M. E. Welland. 2013. alpha-Synuclein senses lipid packing defects and induces lateral expansion of lipids leading to membrane remodeling. *J. Biol. Chem.* 288:20883–20895. https://doi.org/10.1074/jbc.M113.478297.

22. Nuscher, B., F. Kamp, …, K. Beyer. 2004. Alpha-synuclein has a high affinity for packing defects in a bilayer membrane: a thermodynamics study. *J. Biol. Chem.* 279:21966–21975. https://doi.org/10.1074/jbc.M401076200.

23. Johnson, D. H., O. H. Kou, …, W. F. Zeno. 2025. Lipid packing defects are necessary and sufficient for membrane binding of alpha-synuclein. *Commun. Biol.* 8:1179. https://doi.org/10.1038/s42003-025-08622-7.

24. Kubo, S. i., V. M. Nemani, …, D. L. Fortin. 2005. A combinatorial code for the interaction of alpha-synuclein with membranes. *J. Biol. Chem.* 280:31664–31672. https://doi.org/10.1074/jbc.M504894200.

25. Jo, E., J. McLaurin, …, P. E. Fraser. 2000. alpha-Synuclein membrane interactions and lipid specificity. *J. Biol. Chem.* 275:34328–34334. https://doi.org/10.1074/jbc.M004345200.

26. Jiang, Z., M. de Messieres, and J. C. Lee. 2013. Membrane remodeling by alpha-synuclein and effects on amyloid formation. *J. Am. Chem. Soc.* 135:15970–15973. https://doi.org/10.1021/ja405993r.

27. Kaur, U., and J. C. Lee. 2021. Membrane Interactions of alpha-Synuclein Probed by Neutrons and Photons. *Acc. Chem. Res.* 54:302–310. https://doi.org/10.1021/acs.accounts.0c00453.

28. Sciacca, M. F., F. Lolicato, …, C. La Rosa. 2020. Lipid-Chaperone Hypothesis: A Common Molecular Mechanism of Membrane Disruption by Intrinsically Disordered Proteins. *ACS Chem. Neurosci.* 11:4336–4350. https://doi.org/10.1021/acschemneuro.0c00588.

29. Kamal, J. K. A., L. Zhao, and A. H. Zewail. 2004. Ultrafast hydration dynamics in protein unfolding: human serum albumin. *Proc. Natl. Acad. Sci. USA.* 101:13411–13416. https://doi.org/10.1073/pnas.0405724101.

30. Krishnan, R., and S. L. Lindquist. 2005. Structural insights into a yeast prion illuminate nucleation and strain diversity. *Nature.* 435:765–772. https://doi.org/10.1038/nature03679.

31. Sun, Y., L. Breydo, …, I. V. Baskakov. 2007. Site-specific conformational studies of prion protein (PrP) amyloid fibrils revealed two cooperative folding domains within amyloid structure. *J. Biol. Chem.* 282:9090–9097. https://doi.org/10.1074/jbc.M608623200.

32. Ohgita, T., N. Namba, …, H. Saito. 2022. Mechanisms of enhanced aggregation and fibril formation of Parkinson's disease-related variants of alpha-synuclein. *Sci. Rep.* 12:6770. https://doi.org/10.1038/s41598-022-10789-6.

33. Ohgita, T., H. Kono, …, H. Saito. 2023. Intramolecular interaction kinetically regulates fibril formation by human and mouse alpha-synuclein. *Sci. Rep.* 13:10885. https://doi.org/10.1038/s41598-023-38070-4.

34. Spanedda, M. V., M. De Giorgi, …, B. Frisch. 2023. Click Chemistry for Liposome Surface Modification. *Methods Mol. Biol.* 2622:173–189. https://doi.org/10.1007/978-1-0716-2954-3_15.

35. Antimisiaris, S. G. 2023. Preparation of DRV Liposomes. *Methods Mol. Biol.* 2622:21–47. https://doi.org/10.1007/978-1-0716-2954-3_2.

36. Maltseva, S., D. Kerr, …, K. Y. C. Lee. 2024. Parkinson's disease-associated mutations in alpha-synuclein alters its lipid-bound state. *Biophys. J.* 123:1610–1619. https://doi.org/10.1016/j.bpj.2024.05.002.

37. Terakawa, M. S., Y. H. Lee, …, Y. Goto. 2018. Membrane-induced initial structure of alpha-synuclein control its amyloidogenesis on model membranes. *Biochim. Biophys. Acta. Biomembr.* 1860:757–766. https://doi.org/10.1016/j.bbamem.2017.12.011.

38. Meade, R. M., S. G. Allen, …, J. M. Mason. 2023. An N-terminal alpha-synuclein fragment binds lipid vesicles to modulate lipid-induced aggregation. *Cell Rep. Phys. Sci.* 4:101563. https://doi.org/10.1016/j.xcrp.2023.101563.

39. Fusco, G., A. De Simone, …, C. M. Dobson. 2016. Structural Ensembles of Membrane-bound alpha-Synuclein Reveal the Molecular Determinants of Synaptic Vesicle Affinity. *Sci. Rep.* 6:27125. https://doi.org/10.1038/srep27125.

40. Iyer, A., S. J. Roeters, …, V. Subramaniam. 2016. The Impact of N-terminal Acetylation of alpha-Synuclein on Phospholipid Membrane Binding and Fibril Structure. *J. Biol. Chem.* 291:21110–21122. https://doi.org/10.1074/jbc.M116.726612.

41. Navarro-Paya, C., M. Sanz-Hernandez, and A. De Simone. 2022. Plasticity of Membrane Binding by the Central Region of alpha-Synuclein. *Front. Mol. Biosci.* 9:857217. https://doi.org/10.3389/fmolb.2022.857217.

42. Doherty, C. P. A., S. M. Ulamec, …, D. J. Brockwell. 2020. A short motif in the N-terminal region of alpha-synuclein is critical for both aggregation and function. *Nat. Struct. Mol. Biol.* 27:249–259. https://doi.org/10.1038/s41594-020-0384-x.

43. Rey, J., S. Murail, …, P. Tuffery. 2023. PEP-FOLD4: a pH-dependent force field for peptide structure prediction in aqueous solution. *Nucleic Acids Res.* 51:W432–W437. https://doi.org/10.1093/nar/gkad376.

44. Binette, V., N. Mousseau, and P. Tuffery. 2022. A Generalized Attraction-Repulsion Potential and Revisited Fragment Library Improves PEP-FOLD Peptide Structure Prediction. *J. Chem. Theory Comput.* 18:2720–2736. https://doi.org/10.1021/acs.jctc.1c01293.

45. Bacalum, M., B. Zorilă, and M. Radu. 2013. Fluorescence spectra decomposition by asymmetric functions: Laurdan spectrum revisited. *Anal. Biochem.* 440:123–129. https://doi.org/10.1016/j.ab.2013.05.031.

46. Emelyanenko, V. I. R. ,Y. K., O. A. Andreev, and E. A. Burstein. 2000. Log-normal component analysis of fluorescence spectra of prodan and acrylodan bound to proteins. *Biophysics.* 45:207–219.

47. Wearsch, P. A., L. Voglino, and C. V. Nicchitta. 1998. Structural transitions accompanying the activation of peptide binding to the endoplasmic reticulum Hsp90 chaperone GRP94. *Biochemistry.* 37:5709–5719. https://doi.org/10.1021/bi9801006.

48. Meister, A., and E. Brecht. 1986. Principal component analysis of fluorescence spectra from chloroplasts and light-harvesting chlorophyll a/b-protein complex from Vicia faba. *Photobioch. Photobiop.* 11:29–36. https://doi.org/10.1016/s0165-8646(24)00507-5.

49. Al-Soufi, W., M. Novo, and M. Mosquera. 2001. Principal Component Global Analysis of Fluorescence and Absorption Spectra of 2-(2'-Hydroxyphenyl)Benzimidazole. *Appl. Spectrosc.* 55:630–636. https://doi.org/10.1366/0003702011952253.

50. Maltsev, A. S., J. Ying, and A. Bax. 2012. Impact of N-terminal acetylation of alpha-synuclein on its random coil and lipid binding properties. *Biochemistry.* 51:5004–5013. https://doi.org/10.1021/bi300642h.

51. Parasassi, T., E. K. Krasnowska, …, E. Gratton. 1998. Laurdan and Prodan as Polarity-Sensitive Fluorescent Membrane Probes. *J. Fluorescence.* 8:365–373. https://doi.org/10.1023/A:1020528716621.

52. Cheng, D., A. M. Jenner, …, B. Garner. 2011. Lipid pathway alterations in Parkinson's disease primary visual cortex. *PLoS One.* 6:e17299. https://doi.org/10.1371/journal.pone.0017299.
53. Schepers, J., T. Löser, and C. Behl. 2024. Lipids and alpha-Synuclein: adding further variables to the equation. *Front. Mol. Biosci.* 11:1455817. https://doi.org/10.3389/fmolb.2024.1455817.
54. Wang, S., S. Zhang, …, S. N. Witt. 2014. Phosphatidylethanolamine deficiency disrupts alpha-synuclein homeostasis in yeast and worm models of Parkinson disease. *Proc. Natl. Acad. Sci. USA.* 111:E3976–E3985. https://doi.org/10.1073/pnas.1411694111.
55. Tan, L. Y., K. H. Tang, …, S. Jung. 2022. alpha-Synuclein at the Presynaptic Axon Terminal as a Double-Edged Sword. *Biomolecules.* 12:507. https://doi.org/10.3390/biom12040507.
56. Liu, J., B. Bu, …, B. Ji. 2021. Membrane packing defects in synaptic vesicles recruit complexin and synuclein. *Phys. Chem. Chem. Phys.* 23:2117–2125. https://doi.org/10.1039/d0cp03546g.
57. Fusco, G., T. Pape, …, A. De Simone. 2016. Structural basis of synaptic vesicle assembly promoted by alpha-synuclein. *Nat. Commun.* 7:12563. https://doi.org/10.1038/ncomms12563.
58. Fusco, G., S. W. Chen, …, A. De Simone. 2017. Structural basis of membrane disruption and cellular toxicity by alpha-synuclein oligomers. *Science.* 358:1440–1443. https://doi.org/10.1126/science.aan6160.
59. Imaura, R., Y. Kawata, and K. Matsuo. 2024. Salt-Induced Hydrophobic C-Terminal Region of alpha-Synuclein Triggers Its Fibrillation under the Mimic Physiologic Condition. *Langmuir.* 40:20537–20549. https://doi.org/10.1021/acs.langmuir.4c02178.
60. Lautenschlager, J., A. D. Stephens, …, G. S. K. Schierle. 2018. C-terminal calcium binding of alpha-synuclein modulates synaptic vesicle interaction. *Nat. Commun.* 9:712. https://doi.org/10.1038/s41467-018-03111-4.

**Biophysical Journal, Volume *125***

## Supplemental information

# Phosphatidylethanolamine modulates α-synuclein membrane-binding behavior

**Norihiro Namba, Shiori Ariyoshi, Honori Shiroshita, Norihisa Yoshimura, Takashi Ohgita, Shinya Oishi, and Hiroyuki Saito**

## Supporting Information

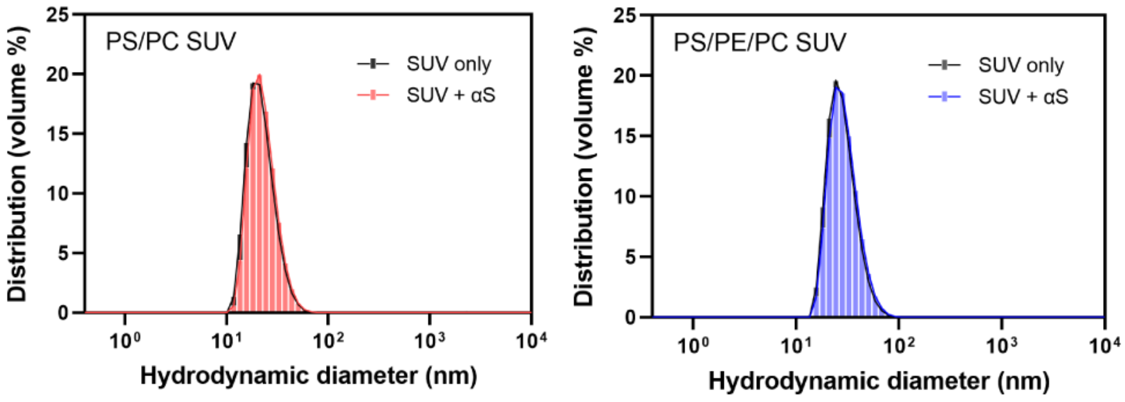


**Figure S1. Effect of α-synuclein (αS) addition on particle size distribution of small unilamellar vesicles (SUVs).** Dynamic light scattering measurements were performed using a Zetasizer Nano ZS (Malvern) to assess the size distribution of SUVs (phospholipid concentration was 500 μg/mL) in the absence and presence of αS (25 μg/mL). Left panel, PS/PC (3/7) SUVs; Right panel, PS/PE/PC (3/5/2) SUVs. PE, phosphatidylethanolamine; PC, phosphatidylcholine; PS, phosphatidylserine. All data represent the average of three independent measurements.

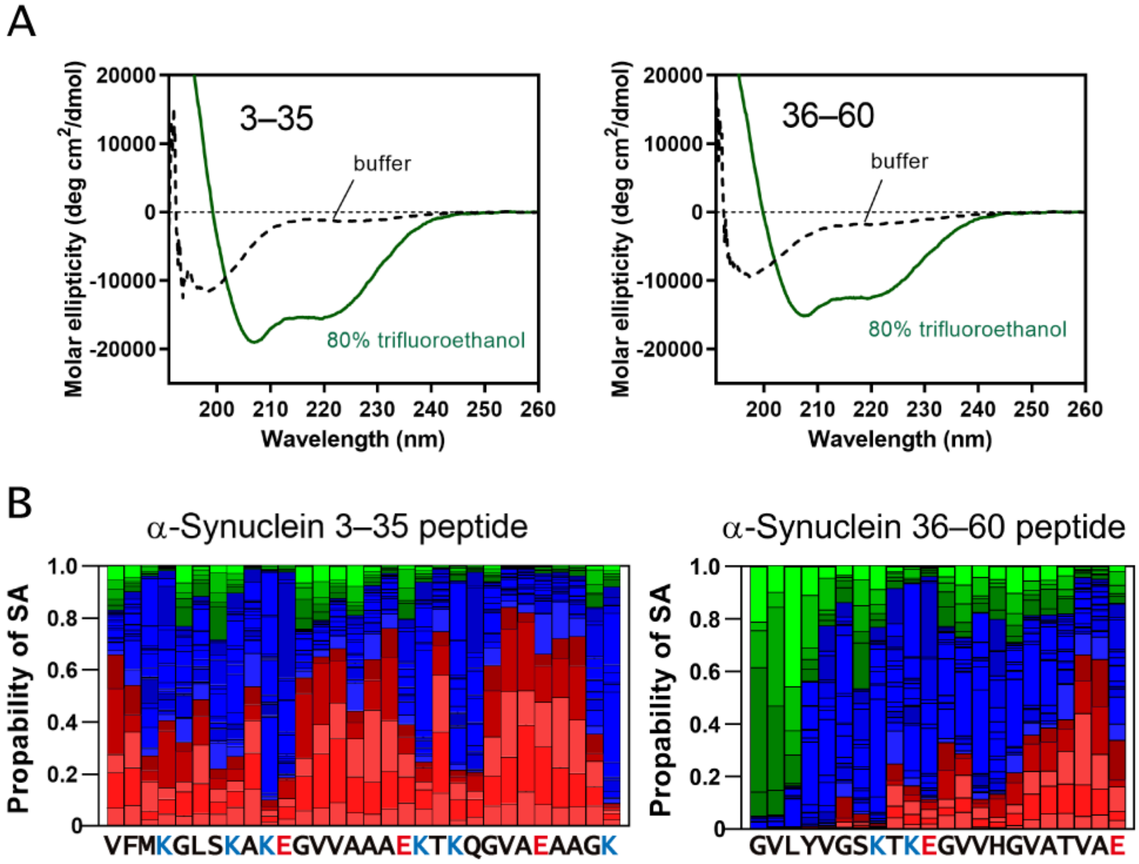


**Figure S2. Secondary structure analysis of αS N-terminal peptides.** (A) Far-UV circular dichroism (CD) spectra of αS peptides corresponding to residues 3–35 and 36–60 (10 μM) in 80% (v/v) trifluoroethanol/20 mM phosphate buffer (50 mM NaCl, pH 7.4). The spectra indicate their α-helical formation propensity. All data represent the average of at least two independent measurements. (B) Predicted secondary structural propensities of αS 3–35 and 36–60 peptides using the PEP-FOLD4 server (https://bioserv.rpbs.univ-paris-diderot.fr/services/PEP-FOLD4/). SA, structural alphabet. Red, blue, and green bars represent helical, coil, and extended structures, respectively.

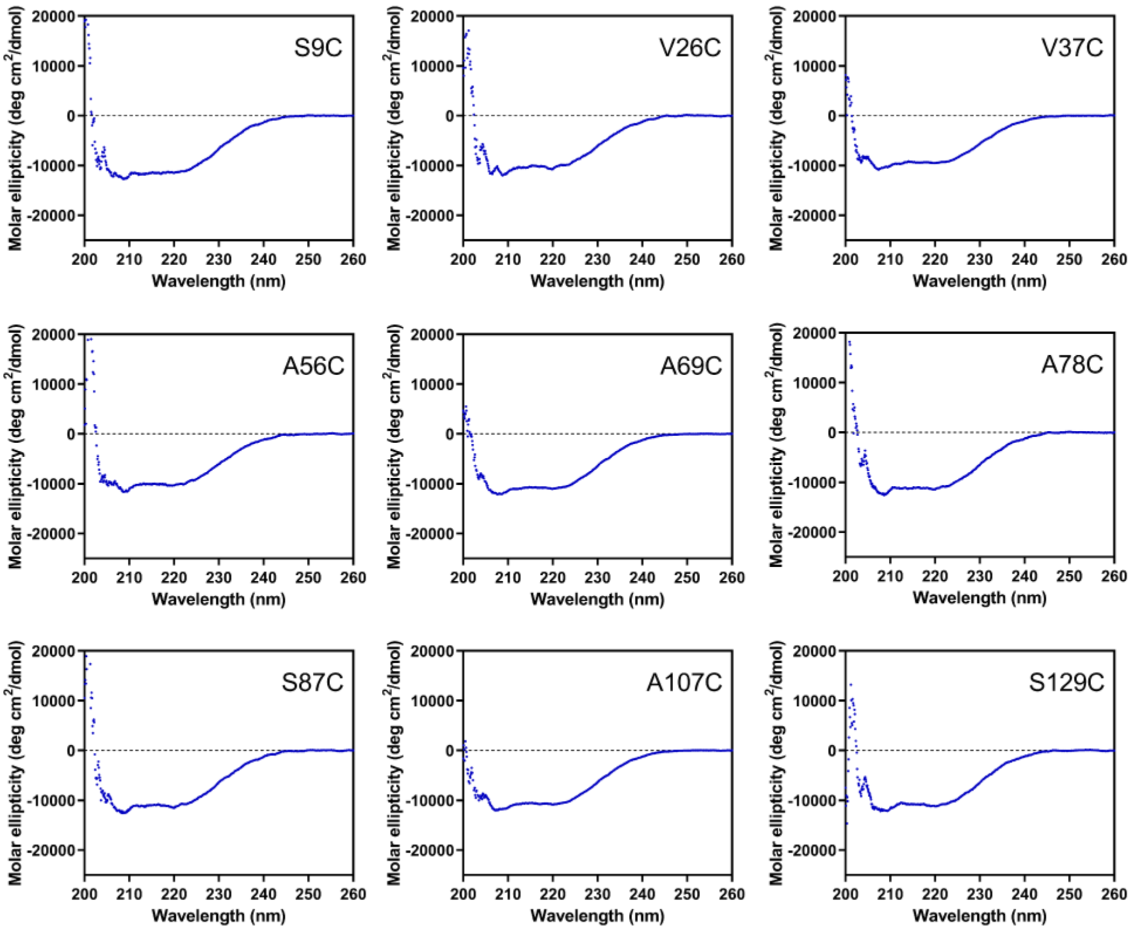


**Figure S3. Far-UV CD spectra of Cys-substituted variants of αS.** Far-UV CD spectra of each αS variant (50 µg/mL) were recorded in the presence of PS/PC (3/7) SUVs (phospholipid concentration was 1 mg/mL) in 20 mM phosphate buffer (150 mM NaCl, pH 7.4) using a JASCO J-1500 spectropolarimeter.

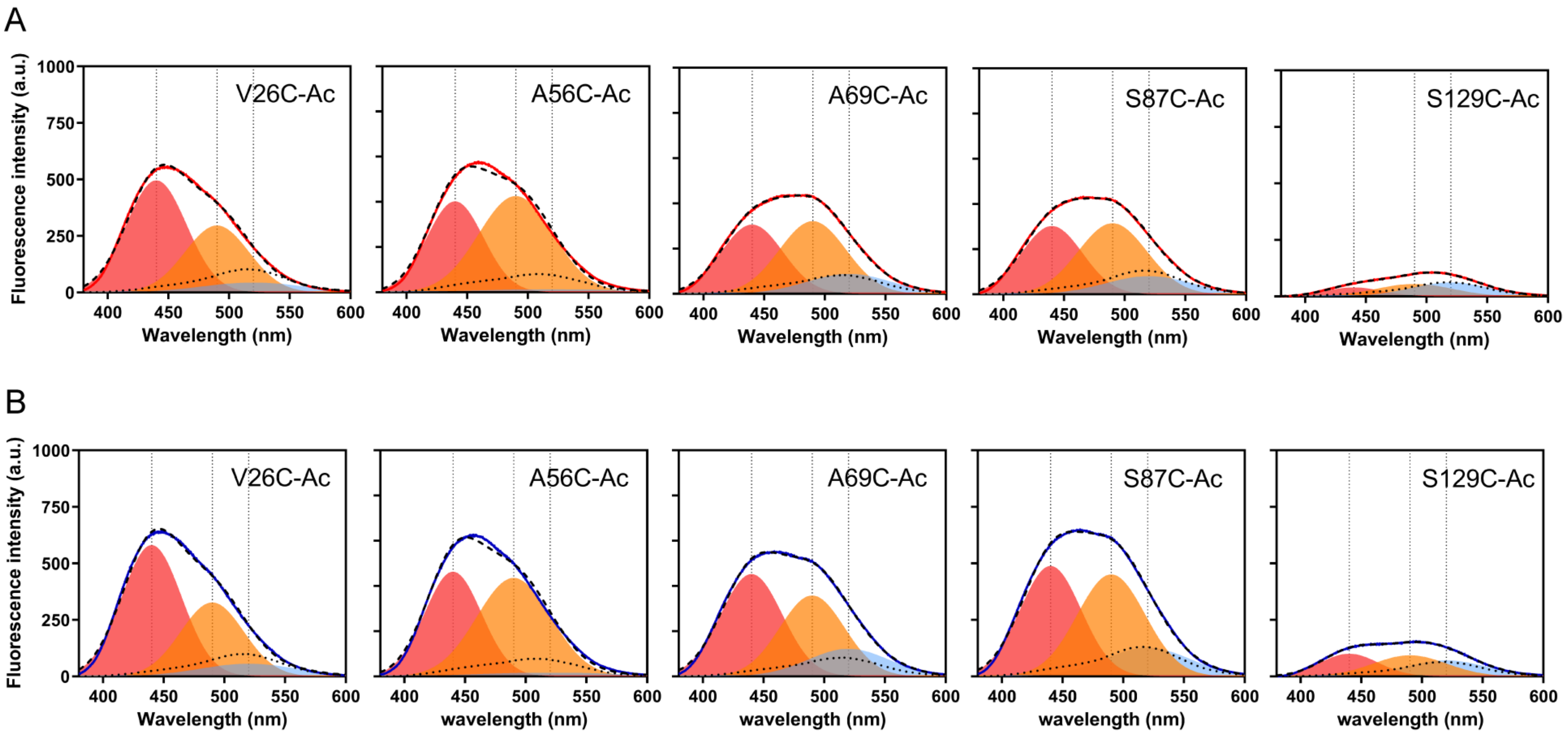


**Figure S4. Gaussian deconvolution of acrylodan (Ac) fluorescence spectra of Ac-labelled αS variants.** Ac fluorescence spectra of αS variants (25 μg/mL) were recorded in the presence of PS/PC (3/7) SUVs (A) and PS/PE/PC (3/5/2) SUVs (B) in 20 mM phosphate buffer (150 mM NaCl, pH 7.4) using an F-7000 fluorescence spectrophotometer (Hitachi High-Tech). The phospholipid concentration was 500 μg/mL. Spectra were deconvoluted into three Gaussian components centered at 440 nm (red), 490 nm (orange), and 520 nm (blue) using the least square method. All data represent the average of at least three independent measurements.

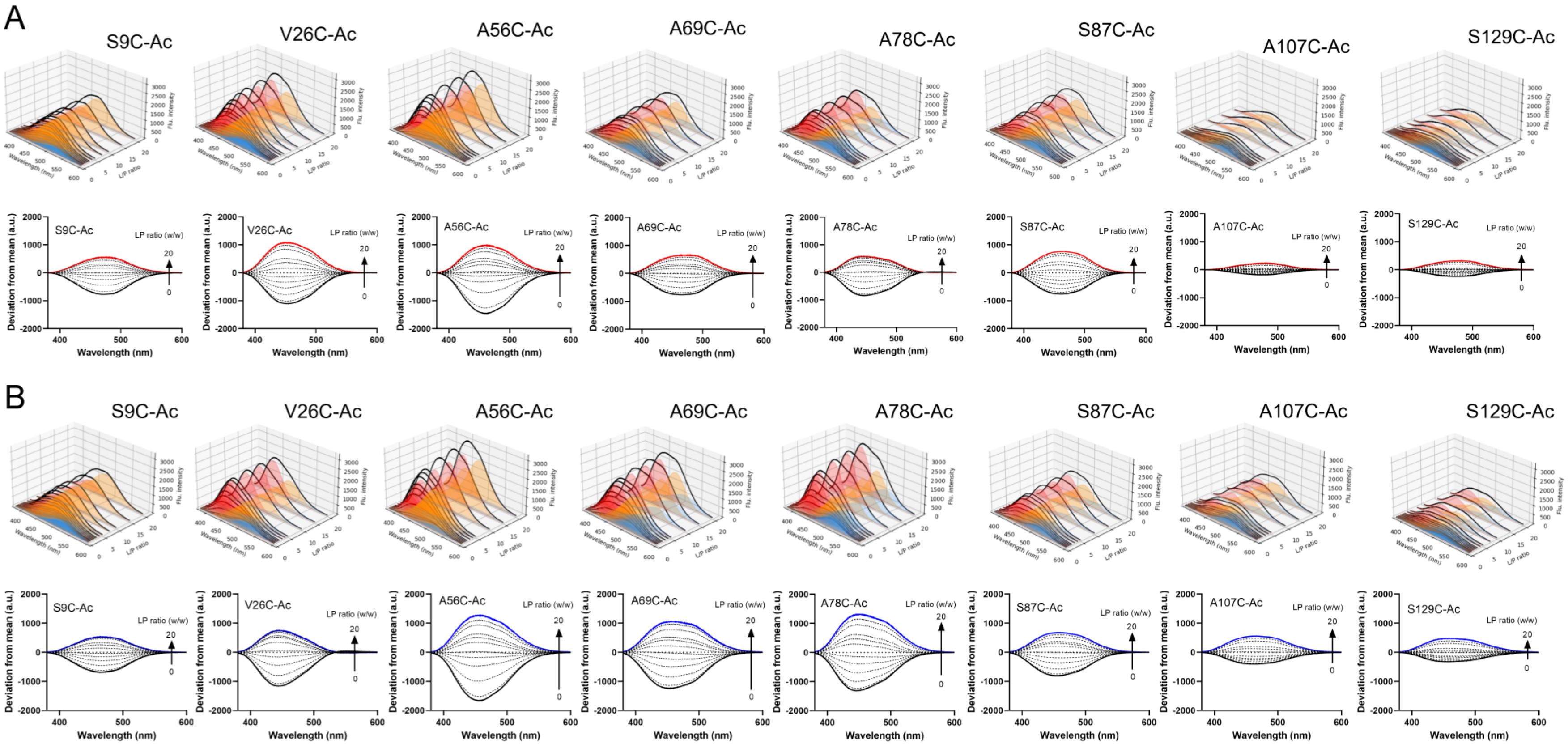


**Figure S5. Ac fluorescence spectra of αS variants labeled at individual residues with varying phospholipid-to-protein ratios.** Spectra were recorded in the presence of PS/PC (3/7) SUVs (A) and PS/PE/PC (3/5/2) SUVs (B) in 20 mM phosphate buffer (150 mM NaCl, pH 7.4). Upper panels, Ac fluorescence spectra of each Ac-labelled αS variant at different phospholipid-to-protein ratios. Lower panels, Mean-centered spectra used for principal component analysis (PCA). The measurements were performed at least twice for each experimental condition.

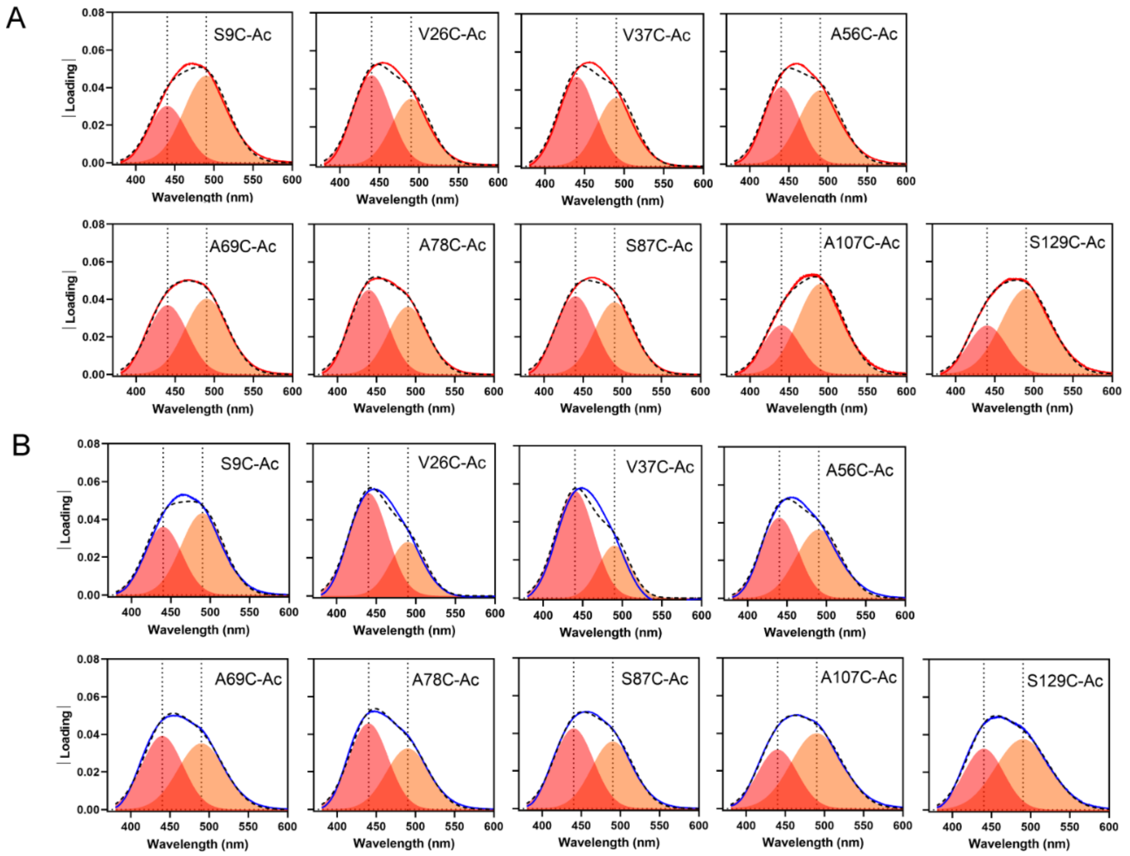


**Figure S6. Gaussian deconvolution of first principal component (PC1) loading spectra of Ac-labeled αS variants.** PC1 loading spectra were derived using PCA of mean-centered Ac fluorescence spectra (see Figure S5), obtained under conditions using SUVs composed of PS/PC (3/7) (A) and PS/PE/PC (3/5/2) (B). Dotted and solid lines represent PC1 loading spectra and the results of Gaussian deconvolution, respectively. Red- and orange-shaded areas correspond to Gaussian components with maxima at 440 nm and 490 nm, respectively.

**Table S1. Contribution ratios of the top five components derived using PCA.** The table presents a summary of the proportion of total variance (normalized to 1.0) explained by the first five principal components (PC1–PC5) obtained using PCA of Ac-fluorescence spectra. PC, principal component.

**PS/PC (3/7)**

| | S9C | V26C | V37C | A56C | A69C | A78C | S87C | A107C | S129C |
|---|---|---|---|---|---|---|---|---|---|
| **PC1** | 0.9998 | 0.9994 | 0.9994 | 0.9994 | 0.9988 | 0.9992 | 0.9996 | 0.9993 | 0.9996 |
| **PC2** | 0.0001 | 0.0004 | 0.0005 | 0.0004 | 0.0011 | 0.0008 | 0.0004 | 0.0003 | 0.0003 |
| **PC3** | 1.9E–5 | 0.0001 | 7.1E–5 | 0.0002 | 9.5E–6 | 2.7E–5 | 4.2E–6 | 6.9E–5 | 3.7E–5 |
| **PC4** | 1.1E–5 | 5.5E–6 | 5.6E–6 | 5.5E–6 | 8.8E–6 | 5.4E–6 | 2.6E–6 | 5.9E–5 | 2.9E–5 |
| **PC5** | 9.2E–6 | 4.7E–6 | 4.1E–6 | 4.8E–6 | 7.5E–6 | 5.0E–6 | 1.6E–6 | 4.6E–5 | 2.2E–5 |

**PS/PE/PC (3/5/2)**

| | S9C | V26C | V37C | A56C | A69C | A78C | S87C | A107C | S129C |
|---|---|---|---|---|---|---|---|---|---|
| **PC1** | 0.9997 | 0.9994 | 0.9993 | 0.9989 | 0.9976 | 0.9974 | 0.9989 | 0.9998 | 0.9998 |
| **PC2** | 0.0002 | 0.0006 | 0.0006 | 0.0010 | 0.0024 | 0.0026 | 0.0011 | 0.0001 | 0.0002 |
| **PC3** | 1.7E–5 | 1.4E–5 | 9.8E–6 | 0.0001 | 7.6E–6 | 7.4E–6 | 3.5E–6 | 2.6E–5 | 1.9E–5 |
| **PC4** | 1.2E–5 | 7.0E–6 | 7.1E–6 | 4.2E–6 | 6.3E–6 | 4.4E–6 | 2.4E–6 | 5.9E–6 | 1.5E–5 |
| **PC5** | 1.0E–5 | 5.9E–6 | 6.0E–6 | 3.7E–6 | 5.7E–6 | 3.4E–6 | 1.7E–6 | 3.3E–6 | 1.3E–5 |
